# High self-selection of Ukrainian refugees into Europe: Evidence from Kraków and Vienna

**DOI:** 10.1371/journal.pone.0279783

**Published:** 2023-12-20

**Authors:** Judith Kohlenberger, Isabella Buber-Ennser, Konrad Pędziwiatr, Bernhard Rengs, Ingrid Setz, Jan Brzozowski, Bernhard Riederer, Olena Tarasiuk, Ekaterina Pronizius

**Affiliations:** 1 Department of Socioeconomics, Vienna University of Economics and Business, Vienna, Austria; 2 Vienna Institute of Demography (OeAW), Wittgenstein Centre (IIASA, OeAW, University of Vienna), Vienna, Austria; 3 Department of International Affairs and Centre for Advanced Studies of Population and Religion, Cracow University of Economics, Kraków, Poland; 4 Institute of European Studies, Jagiellonian University, Kraków, Poland; 5 Department of Sociology, University of Vienna, Vienna, Austria; 6 Population and Just Societies Program, International Institute for Applied Systems Analysis, Laxenburg, Austria; 7 Department of Cognition, Emotion, and Methods in Psychology, University of Vienna, Vienna, Austria; Emory University, UNITED STATES

## Abstract

Almost eight million Ukrainians have fled their country to escape the Russian full-scale invasion. To provide empirical evidence on how beneficiaries of temporary protection who reside in the immediate proximity of Ukraine differ from those who went further and reside in Western European countries, two large-scale rapid-response surveys were conducted in Kraków, Poland, and Vienna, Austria, in spring 2022. Data include information on socio-demographic characteristics, human capital, and return intentions of 472 and 1,094 adult Ukrainian refugees in Poland and Austria, respectively. Contributing to the growing empirical evidence on consistent assortative patterns in refugee inflows into Europe, our findings show that regularities in patterns of self-selection also occur in forced migration contexts where legal routes to safety apply. According to the analysed convenience sample, a tentative conclusion is that the further Ukrainian refugees moved to the West, the more self-selected they tend to be in the key dimensions of formal educational attainment, previous employment, language skills, and urbanity. Results indicate that willingness to stay in Kraków is significantly lower than willingness to remain in Vienna. This suggests that public financial support and living conditions, rather than diaspora networks, are decisive factors in shaping the decision to stay, move to another location or return to Ukraine. The aim to start a new life elsewhere may drive the motivation to choose a more distant destination instead of a neighboring country that allows to return rather quickly. Host countries should be aware of these specific characteristics of their refugee populations and adapt their integration policies accordingly.

## Introduction

The Russian military invasion of Ukraine, which began on February 24, 2022, led to the largest forced migration flows in Europe since WWII and one of the largest forced displacement crises in the world today. Reports from the United Nations High Commissioner for Refugees (UNHCR) show that more than one year after the onset of the Russian invasion, nearly one-third of Ukrainians (over 13 million people) have been forced from their homes and that more than eight million have fled the country and are residing mainly in European countries [1, 2]. Neighbouring countries such as Poland, Slovakia, and Hungary, but also Western European nations, most notably Germany [3, 4] and Austria [5], are hosting refugees, providing shelter, access to healthcare, education, as well as everyday support infrastructure. According to the UNHCR, Poland was hosting over 1.6 million Ukrainian refugees as of May 2023 [2], distributed all across the country with a particularly high concentration in the twelve largest Polish cities. In Austria, national statistics report that roughly 70,000 Ukrainian refugees have been registered as of January 2023, with the majority being located in the country’s capital Vienna [6].

Due to the prompt first-time activation of the Temporary Protection Directive for displaced persons from outside EU territory [7], Ukrainians hosted in EU member states are guaranteed immediate access to the labour market and social assistance, as well as a residence permit for one year, which can be extended for a further two years. However, with the continuation of the Russian war of aggression and the deliberate destruction of Ukrainian infrastructure, it has become increasingly clear that return to Ukraine will not be an immediate option for a sizable number of refugees. For them, their stay in the host countries might become permanent beyond the duration of temporary protection measures.

Little is known to date about actual return and staying intentions of Ukrainian refugees, especially when discussing different countries of arrival, multiple possible destinations or intended onward movement within the Schengen area (as permitted by the temporary protection status). Similarly, much remains to be ascertained about their specific needs and resources and the overall socio-demographic composition of the displaced population in the respective host countries. So far, first insights point to a predominantly female, young- to middle-aged, highly qualified displaced population [8–10]. Yet, more detailed and country-specific data are crucial for government agencies, humanitarian organisations, and civil society in order to adapt integration and potential return assistance to the specific needs of refugees.

Our large-scale cross-sectional surveys in Poland and Austria address this research gap by providing the first reliable data on the Ukrainian displaced population in two host countries heavily affected by recent refugee inflows. We want to provide empirical evidence on how beneficiaries of temporary protection who reside in the immediate proximity of the Ukrainian border, such as Poland, differ from those who travelled further and are now staying in Western European countries, such as Austria. Specifically, we aim to address the key question of who the Ukrainian refugees are: What are their educational attainment levels, qualifications, and skills to support labour market integration in the host countries? Second, what are their intentions to stay and/or to return, or to reunite with family within the host country? We contextualise our findings within a growing body of evidence of refugees’ high degree of self-selection into Europe, most recently demonstrated during the 2015 inflows from Syria, Afghanistan and Iraq [11, 12].

## Background

### War in Ukraine

The Russian full-scale invasion, which began on February 24, follows over eight years of war in Donbas and the occupation of Crimea which produced around 1.5 million internally displaces persons prior to [13, 14]. Since the outbreak of the war, millions of Ukrainians were forced to leave their homes seeking safety and protection in the western regions of Ukraine or beyond the borders. As a result, by early 2023, the number of IDPs has increased by more than three times reaching numbers between 4.9 and 5.4 million people [15, 16]. Many of IDPs were forced to flee the war for a second time in their lives. Over 8.2 million Ukrainian refugees have been recorded across Europe, including 5.1 million having registered for temporary protection or similar national protection schemes [15]. The highest number of Ukrainians registered for temporary protection are reported from Poland, but a sizeable number of refugees has also reached Austria.

### Polish country context

The beginning of the war in Ukraine in 2014 and the increasing number of Ukrainians arriving in Poland sped up the transformation of Poland from a country of emigration to one of immigration. Over two million Poles had left the country following the collapse of communism in search of work and better living conditions, however, after 2014, the number of Poles residing abroad had stabilised. At the same time, many Ukrainians began arriving, as the occupation of Crimea and the war in Donbas pushed an increasing number of Ukrainians to Poland rather than to Russia [17]. Only an insignificant number of persons seeking international protection used Poland as a transit country into Western Europe or beyond. An exploratory study carried out by Statistics Poland in 2020 suggests that the Ukrainian population in Poland could be as large as 1.3 million people. This considerable size of the Ukrainian diaspora in Poland prior to 2022 is particularly noteworthy since it has been playing a crucial role in all the reception and integration efforts. Contrary to asylum seekers from the Middle East and other parts of the world who are strongly racialised, pushed back and clustered at the Belarusian border [18, 19], Ukrainians have been “welcomed” refugees and public attitudes towards them have been largely favourable [10, 20].

### Austrian country context

In contrast to Poland, the Ukrainian diaspora in Austria at the beginning of 2022 was rather small, amounting to around 16,500 persons. By July 1, however, it had increased significantly to around 80,000 registered persons [21], with a large share residing in the Austrian capital, Vienna (40%). Even before the Russian invasion, immigration was already an important aspect of Austrian society. In 2022, roughly two out of ten persons living in Austria and almost four out of ten persons living in Vienna were born abroad [21]. In the past, the country received high numbers of asylum seekers due to political unrest and armed conflicts in neighbouring Eastern European countries (e.g., Hungary, Czechoslovakia, Yugoslavia) [22, 23]. In addition, displaced persons from the Middle East and Asia increasingly arrived in Austria at the beginning of the 21^st^ century, and in 2015, the country was the fourth largest recipient of asylum seekers in Europe [24], originating mainly from Syria and Afghanistan.

## Literature review

Early insights from online surveys and qualitative interviews suggest high self-selection among Ukrainian refugees in Europe. Overall, surveys corroborate initial impressions that Ukrainian refugees are predominantly female, in their mid- to late 30s and are characterised by (very) high levels of education [25, 26]. Accordingly, Pötzschke and Weiß [9] as well as Brücker and colleagues [4] find that women strongly outnumber men in both Germany and Poland. Using targeted advertisements on Facebook and Instagram during the second half of April, the survey by Pötzschke and Weiß [9] suggests notable differences between the Ukrainian population hosted in the two countries. While in both countries, a clear majority were hosted by private individuals, the share of respondents who stayed either with friends and family or with other private citizens was somewhat higher in Germany than in Poland. In contrast, significantly more of those surveyed in Poland stayed in hotels or hostels than in Germany. This seems to be, among other factors, due to the Polish government’s policy to open student dorms to Ukrainian refugees [27]. However, dorms became increasingly less available with the return of students from their spring and summer breaks. The difference could also point to different levels of social capital of the displaced populations in Poland and Germany, respectively, with the latter having more substantial social networks to rely on for accommodation and support. Similarly, available networks might have influenced the choice of host country, as indicated by previous research [28–30].

Urbanity is another key aspect of self-selection of Ukrainian respondents [31]. Those who remained in Poland were predominantly coming from neighbouring regions in Western Ukraine, such as Lviv. In the German sample, a much higher percentage of refugees stems from Kiev (19% versus 9% in the Polish sample). Finally, another key determinant of self-selection is human capital. Again, Ukrainian refugees in Germany showed higher educational attainment than those sampled in Poland, with 64.2% having obtained a tertiary educational degree (Bachelor’s degree or higher), versus 55.5% in the Polish sample.

Other studies also confirm the high self-selection among Ukrainian survey participants. A large-scale online survey by the EUAA and the OECD finds that 73% have a Bachelor’s or Master’s degree and 77% were employed before the outbreak of the war [30]. Furthermore, an online survey by the University of Southampton [32] among IDPs in several European host countries found that the majority of Ukrainian refugees report average or good levels of overall health, while 20% of IDPs reported poor or very poor health. This may reflect the ongoing toll that the war has taken on the health of the remaining population, but also suggest self-selection of those able to leave the country [33, 34].

Educational self-selection can be observed to increase with growing distance from Ukraine, as does self-rated health: While among IDPs, 67% hold a tertiary degree, this is true for 69% of Ukrainians in Poland and 78% in Germany [32]. This is corroborated by the EUAA and OECD survey [30], with refugees in Germany (34%) and Sweden (36%) more often working in managerial positions than those in Romania (21%) and Czechia (20%). In addition, self-selection may impact and, in turn, be dependent on return intentions. While in the Netherlands, Estonia, the UK, and Germany, Ukrainians were most sceptical about being able to return in the foreseeable future [32], respondents in closer proximity to Ukraine, namely in Romania, Poland, and Bulgaria, were more optimistic about a potential return. Similarly, one in four respondents stated that they had not yet arrived at their final destination and might again move in the next few weeks.

For Finland, an online survey conducted by the Ministry of the Interior in June and July 2022 among 2,000 respondents finds that refugees came from areas most affected by the war, in particular the oblasts Kharkiv, Kyiv, and Donetsk, and had earlier ties in Finland, including friends, relatives, or colleagues [35]. Again, educational self-selection was high, with every second respondent having a university degree at Masters’ level. Accordingly, one third of respondents had worked in managerial positions or were skilled professionals or entrepreneurs. Similarly, English language skills were stated by one in three respondents.

The Finish survey also paints a nuanced picture of Ukrainians’ return intentions. While the majority of respondents (40%) had not yet decided whether they wanted to return to Ukraine, one out of three had decided to return to Ukraine when the war is over. Roughly a quarter of surveyed Ukrainians did not plan to return. Factors influencing a desire to stay in Finland were employment (70%), continuation of the war (66%), children’s wellbeing (50%), housing (32%), and language skills (28%).

An online survey conducted in May 2022 among female Ukrainians in Austria finds similarly high educational levels, with 72% of women having a university degree [5]. Consequently, refugees had qualifications for working in education (21%), finance (19%), and administrative work (16%). Nine out of ten respondents intend to find a job in Austria, though caring responsibilities might make full-time employment more difficult. Similar to the findings by Pötzschke and Weiß [9] for Germany, a high share of respondents had social ties in the host country, mostly friends (27%) and family (18%). Furthermore, a quarter of respondents had the financial means to rent their own apartments in Austria. Only 30% had concrete plans to return to Ukraine within the next months.

These results are corroborated by the ongoing UNHCR “Lives on Hold” survey conducted in a mix of phone-based, web-based and face-to-face interviews in most European countries hosting Ukrainian refugees [8]. While the first wave of the survey in May and June 2022 found high return intentions within the next month [40% of respondents, 8], the second wave in August and September 2022 shows a considerable decrease [36]: Only 13% of Ukrainian refugees across all surveyed countries plan to return within the next three months. In view of the fact that one in four Ukrainians already wanted to migrate prior to the war [37], this may suggest a high share of displaced refugees remaining permanently in host countries. Insights from Poland confirm this: Almost 60% of surveyed refugees want to stay until the war is over, while 27% do not want to return at all [38]. Self-selection is also observed in terms of income and earnings, with 31% reporting savings and 12% transfers from friends or relatives in Ukraine, as well as, again, in terms of education, with 70% of respondents completed university or higher studies.

For all of the above studies, coverage errors and response biases are to be considered when interpreting results. In particular, this applies to educational bias, as higher educated respondents have been shown more likely to participate in social science surveys [39–41]. Similarly, participation in the above surveys varied considerably by age and gender, while overall health needs might have been under-estimated. In general, rapid-response surveys among refugees and vulnerable migrants, conducted in dynamic, high-stakes settings or online, are unlikely to be representative of the overall displaced population in the respective countries [42–44].

Nevertheless, the preliminary insights into the socio-demographic composition of the Ukrainian displaced population in Europe support the growing body of evidence on self-selection of refugees and humanitarian migrants into host countries. For the long summer of migration 2015, Aksoy and Poutvaara [45] find that refugees were positively self-selected with respect to human capital, and both male and female migrants from major conflict countries were positively self-selected in terms of their education. For asylum seekers in Germany, Guichard [46] finds that those from Afghanistan, Iraq, and Syria were more likely to have tertiary education than non-migrants, which was, among other factors, linked to the high costs of forced migration. Buber-Ennser et al. [12] show that Syrian and Afghan refugees’ educational self-selection in Austria is linked to less traditional attitudes toward gender equality and lower levels of religiosity. Similar effects have been shown for previous refugee flows from Argentina and Chile [47] and the Soviet Union [48].

Understanding the selection patterns and return intentions among Ukrainian refugees is of paramount importance from the perspective of Ukraine’s socio-economic and political landscape after the end of the military conflict. Extensive literature on the attitudes of refugees following political stabilization in their home countries suggests that returning individuals can play a crucial role as agents of change [49, 50]. This phenomenon is attributed to the paradoxical "post-traumatic growth" theory, holding that personal growth and development can result from drastic life crises [51]. Therein, individuals who have endured challenging situations, including conflict-related violence, tend to respond positively to these traumatic memories by re-evaluating their lives and building deeper connections with fellow individuals [51]. Such persons are found to exhibit less selfishness and a greater inclination toward collective and pro-social activities [52].

Another area of research stems from interdisciplinary studies carried out by psychologists, anthropologists, political scientists and economists focusing on the exposure to war violence and individuals’ behaviour in post-war times [52–54]. In a meta-analysis based on 16 studies on the impact of war exposure on cooperation (including self-reported behaviour as well as experimental games), Bauer and colleagues [52] find evidence of an increase in various dimensions of cooperation and prosocial behaviour: individuals who have experienced wartime conflict tend to show a positive association with post-war in-group cooperation, an increase in political engagement, in participation in elections and in knowledge of politics [52]. Further, scholars analysed the relations between specific behaviour (e.g., voluntary cooperation) and socio-demographics of refugees [54, 55]. Most recently, a study explored the effects of violence on fairness preferences in Eastern Ukraine, pointing to the short-term destabilizing effects of conflict on prosocial preferences with potential long-term consequences [56].

## Methodology and data

Our analyses draw on two cross-sectional surveys that collected data on Ukrainian displaced persons who arrived in Kraków, Poland (UkrPL) and in Vienna, Austria (UkrAiA), respectively. While both surveys cover a wide range of key topics, our analysis focuses on the socio-demographic characteristics of Ukrainian respondents, their human capital, qualifications, labour market experiences as well as their intentions to return and stay in host countries. The corresponding survey items are based on existing survey instruments, such as the Labour Force Survey, the Generations and Gender Survey, a social survey among refugees arriving in Austria in 2015 [12] as well as a survey on integration of immigrants in Kraków and Malopolska Region [57]. Both surveys aim to provide timely and comparable cross-sectional data of Ukrainian displaced persons arriving in various host countries. Thus, they simultaneously collected data between April and June 2022. The comparative nature makes them a highly suitable data source for our analyses. For detailed information (e.g., on sampling, external and internal validation) see S1 File, for the English questionnaires see S2 and S3 Files, Ukrainian and Russian questionnaires are available upon request.

Each potential participant was provided with an information sheet in their native language, explaining the aims of the survey, emphasizing its voluntary nature, and assuring data protection. Verbal explanations were also offered upon request. Moreover, participants were allowed to review the entire questionnaire before deciding whether to participate. Only persons who explicitly provided verbal consent to one of the interviewers–aided by interpreters—were handed a questionnaire to fill out or provided with an individual one-time QR code to scan. Considering the sensitive nature of the population under study, the provision of contact data, such as phone number or email address, was optional. The vast majority of survey respondents decided on anonymity, making it impossible to directly document their consent while preserving their anonymity. For ethical and legal reasons, only adult participants were accepted, as interviewing minors would require explicit consent from a person with legal parental authority, which could not be obtained anonymously. Furthermore, to eliminate any instances where consent may have been ambiguous, we discarded all questionnaires that were not explicitly returned to our interviewers after completion, as well as any partially filled-in ones. All questionnaires were entered into two distinct databases. The first did not have any fields that could potentially contain any contact information/ partial names etc., ensuring complete anonymity. The second database solely stored contact information but excluded any identifiers or pseudonyms, thus making it impossible to merge these databases and subsequently identify any participant. In addition, all paper sheets containing personal data were physically removed from the questionnaire thereby anonymizing the paper questionnaires, too.

The data collection of both surveys follows convenience sampling. It was collected in numerous locations where Ukrainian refugees assemble to register, arrange documents, or receive help in Kraków, and at the first reception centre in the city of Vienna. The target population comprises Ukrainian refugees aged 18 and older, who intended to stay in the host country, i.e. who were not in transit. We followed a multi-mode approach and collected data via paper and pencil interview (PAPI) and additionally via computer-assisted web interview (CAWI) in Vienna. Considering linguistic and cultural challenges when surveying migrant populations [58, 59], the fieldwork was supported by interpreters (Ukrainian and/or Russian speaking) in addition to the academic supervision and student helpers. Respondents could choose whether to complete the survey in Ukrainian, Russian or English language. The Austrian and Polish survey designs approved by the ethics committees of the Vienna University of Economics and Business and the Cracow University of Economics, respectively. The approval can be obtained upon request.

Our sample consists of 472 interviewed displaced persons in Kraków and 1,094 in Vienna, adding up to a total of 1,566 individuals aged 18 and over. Respondents were on average 39 years old and predominantly female (97% in Kraków and 90% in Vienna). Since both surveys are designed as household survey, proxy information on potential partners and children is collected in addition to the respondents’ information. Overall, we therefore collected data from 1,253 individuals in Kraków and 2,792 in Vienna. Information on partners and children is mainly used for demographics. Subjective information (e.g., intentions) was only collected for respondents themselves. As case numbers for men are often low if partner information is unavailable, parts of the analyses only present figures for all respondents or specifically for women.

## Results

### Demographics

In 2019, Ukraine had a demographic structure similar to the average of the EU-27 countries, characterised by a comparable low share of individuals in younger age cohorts and higher numbers in older cohorts (S1 Fig) [60]. In contrast to the pre-war population, surveyed Ukrainian refugees differ significantly in their demographic characteristics: They are disproportionately female and in young or middle adulthood, or children (S2 Fig). These large gender differences are consistent with previous evidence of Ukrainian refugees’ demographic distribution and reflect martial law, which obliges men between 18 and 60 years to remain in Ukraine for military service [61].

In both host cities, adult women are on average 39 years old and slightly younger than the corresponding male refugee population. Within the surveyed refugee populations (i.e., including respondents as well as their children and partners living in the host country at the time of the interview), a distinction between individuals below age 20 (i.e. children and teenagers), women aged 20–59 years, men aged 20–59 years and persons aged 60 years and more, leads to following shares: 39%, 50%, 7%, and 4% in Kraków and 37%, 45%, 11%, and 7% in Vienna. Therein, children, teenagers, and women of working age amount to 89% of refugees in Kraków and 82% in Vienna, respectively.

Respondents came from all regions in Ukraine, and in the Viennese sample, the share of persons from Kyiv is strikingly high (30% as opposed to 16% in the Kraków sample). The most commonly reported origin of respondents in the Viennese survey after Kyiv were: Kharkivska (8%), Lvivska (7%), Odeska, (7%), Dnipropetrovska (7%), and Donetska oblasts (7%), and in Kraków respectively: Kharkivska (12%), Dnipropetrovska (10%), Donetska (10%), Zaporizka (7%), and Lvivska (5%) oblasts (S3 Fig). Those regions of Ukraine belong to the most economically developed, with the highest GDP and population density before the war (S3 Fig). They also had the highest number of students and numerous higher education institutions. In fact, we found a positive correlation between GDP per capita and respondents’ origin as well as between respondents’ origin and the number of students in the region, the correlation being stronger in the Viennese sample than the one obtained in Kraków (S4 Fig).

Reflecting the time of the field phase, those who left Ukraine soon after the outbreak of the war are strongly represented in our data: Roughly six out of ten had arrived in the host country by early March. Arrivals in the second half of March, April, or May were more frequent in Vienna. As it took an average of three days to arrive in Poland and four days to arrive in Austria, we tentatively conclude that our data contains primarily those refugees coming directly to Austria and only to a low extent secondary migrants (i.e. persons who first fled to neighbouring countries, stayed there for some time and then proceeded to Austria).

In both cities, six out of ten respondents were in a relationship, i.e. they were–at the outbreak of the war–either married or lived in a non-marital union. Divorcees and singles comprised 13% and 22%, only few were widowed (S1 Table). Country differences became evident regarding parenthood in the sense that displaced Ukrainians in Vienna were to a higher extent childless (30% versus 39%). Among parents, the parity distribution was similar, with about half having one child and 38% having two children. Parents had on average 1.6 children. In addition, another 2% of female respondents were expecting a child at the time of the survey.

Numerous families were separated due to external displacement, and information on the partners and children allowed us to assess the family context in the host country. Among respondents in relationships (either married or cohabiting), only some arrived as a couple in the host country or reported that their partner had joined them by the time of the interview. The majority indicated that their partner had stayed in Ukraine, only a small fraction named other countries, and a negligible group either did not know or did not provide their partner’s location (S1 Table). Partnered respondents in Vienna significantly more often reported living with their spouse/partner in the host country than those in Kraków (37% versus 20%). Further analyses revealed no clear pattern for this large proportion of women arriving with their partner in Austria. For example, respondents with three or more children were not more likely to arrive with their partner than those with fewer children (results available on request). Thus, the potential for future family reunification will be dominated by married men joining women, whereas previous refugee inflows to Austria and Europe were mainly about reunification with female partners and children [e.g., 12].

### Education

**Fig 1:**
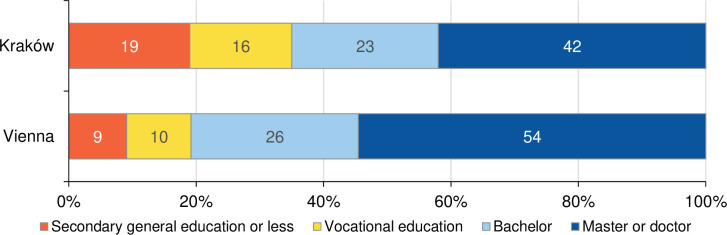
Educational attainment among surveyed refugees, aged 18+ years. Sources: UkrPL and UkrAiA. Note: Test for differences between Kraków and Vienna: Chi^2^(3) = 46.92, p<0.001. 95% confidence intervals for “Secondary general education or less”: Kraków [15; 22]; Vienna [7; 11]; for “Vocational education”: Kraków [13; 20]; Vienna [8; 12]; for “Bachelor”: Kraków [19; 27]; Vienna [23; 29]; for “Master or doctor”: Kraków [37; 46]; Vienna [51; 57].

The educational level of respondents was exceptionally high: About four out of ten respondents in the Kraków sample and five out of ten in the Viennese sample reported a master degree or a doctoral degree, further one out of four had obtained a bachelor’s degree (Fig 1). Vocational training was more common among respondents in Kraków than in Vienna (16% versus 10%). The proportion of respondents holding secondary education or a lower level of education was twice as large in Kraków than in Vienna (19% versus 9%). Our results are thus in line with early insights from the UNHCR [8] or online surveys [9] indicating high levels of education among Ukrainian refugees who participated in surveys across Europe. Further, a positive selection of migrants in terms of education and skills has been widely documented and externally displaced persons tend to have higher skills compared to the general population of their country of origin [45, 62].

The Viennese survey includes a substantial number of interviewed men as well as information on the educational level of the partner, and thus allows differentiation by sex and place of residence. Results show a higher level of education among women, which is in line with the educational attainment of the general population in pre-war Ukraine [63]. Furthermore, the educational level of male partners remaining in Ukraine was similar to that of the surveyed refugee population in the host country.

**Fig 2:**
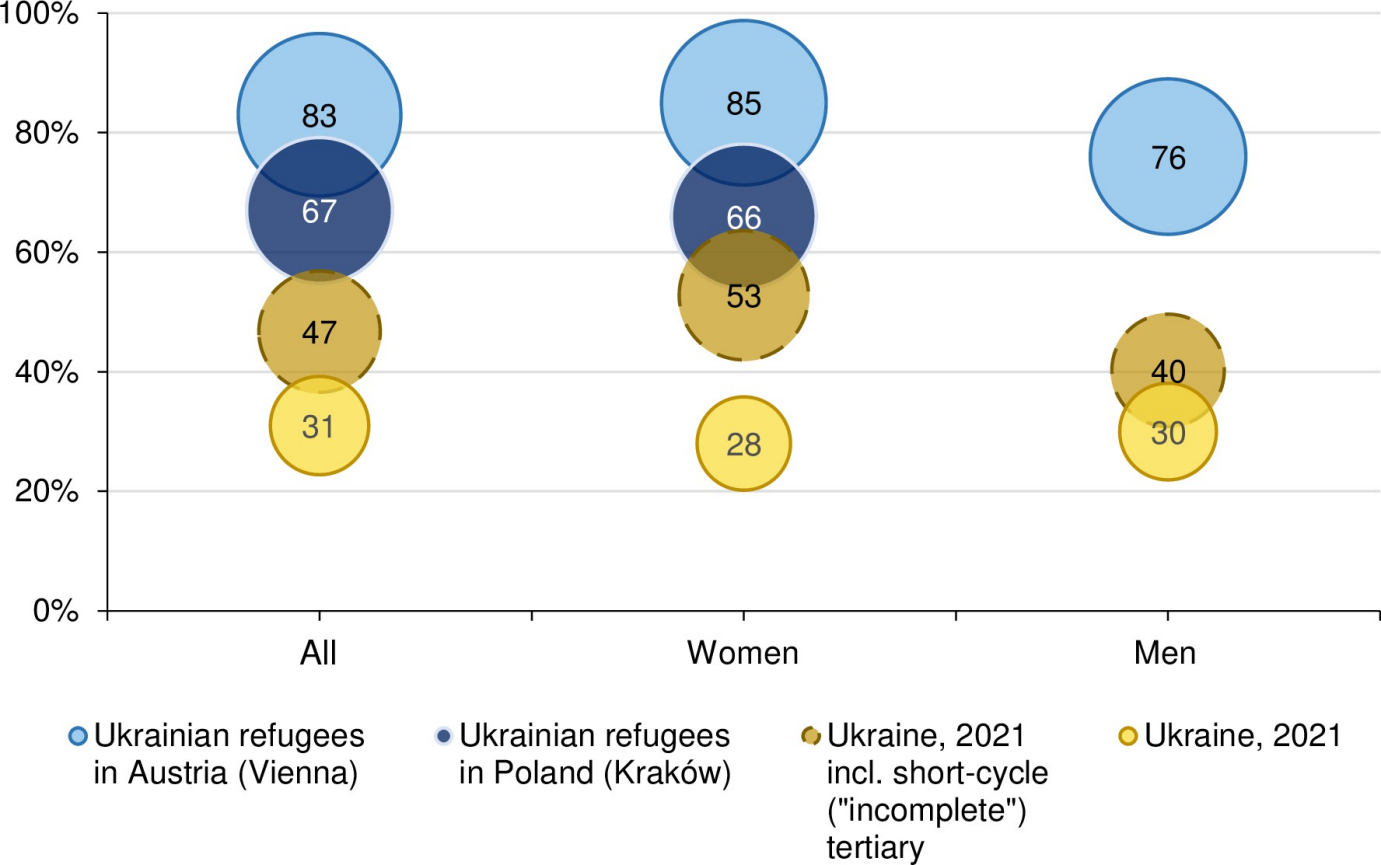
Share of tertiary educated among persons aged 25–64 years in the general population of Ukraine and among respondents in Vienna and Kraków, by sex. Sources: State Statistics Service of Ukraine [63] UkrPL, and UkrAiA. Note: Test for differences between Kraków and Vienna: Chi^2^(1) = 53.01, p<0.001. 95% confidence intervals for “All”: Kraków [62; 71]; Vienna [81; 85]; for “Women”: Kraków [62; 71]; Vienna [70; 82]. Data for Vienna also include partners residing in Austria. Circles with dashed lines include persons with short-cycle tertiary education (so-called “incomplete” tertiary education).

The high selectivity of surveyed refugees becomes evident when compared with the educational distribution in the home country: In 2021, among the Ukrainian population aged 25–64 years, the proportion of those holding a bachelor’s degree or higher tertiary education was 30%. Among surveyed Ukrainian refugees, this share amounts to 67% in Kraków and 83% in Vienna (Fig 2). Thus, the comparison between the two refugee populations reveals a remarkable self-selection in terms of education, which turned out to be more pronounced with increasing distance from the home country, as the share of tertiary educated is much higher among respondents in Austria than in Poland. Educational differences between interviewed refugees in the two cities are highly statistically significant (see notes in Figs 1 and 2). This is consistent with findings on refugees from Syria, showing that those with lower education tended to flee to neighbouring countries while those higher educated were more likely to leave for Europe [11]. Although previous literature on externally displaced persons documented self-selection in terms of education and social status [11, 12, 45], the high share of tertiary educated persons among Ukrainian respondents is striking.

Echoing their high educational attainment, knowledge of foreign languages is pronounced in both samples. Apart from the fact that knowledge of Russian language is almost universal, one third of responding refugees in Poland and two thirds in Austria report to speak English (Chi^2^(1) = 102.93; p<0.001). Skills of the language of the host country were less common (Kraków: 21% report Polish language skills; Vienna: 15% report German language skills) (Chi^2^(1) = 8.44; p = 0.004), but played a role for the active choice of the host country (see results on choice of the host country). In addition, knowledge of other languages (e.g., Hungarian, Polish) were reported by a non-negligible proportion (Vienna: 18%; Kraków: 6%). The skill level was not captured in the surveys and might not be at an advanced level. Nevertheless, being able to adequately communicate in English may prove crucial for participation in the labour market, as Polish and German language skills were only reported by a comparatively low number, communication in the host country might be challenging for many refugees. In Austria, German language courses are provided to asylum seekers and refugees for free, which is crucial for participation and inclusion in social life.

### Employment

Employment can only be accurately assessed for female respondents because the number of male respondents was too small. Most female respondents report work experience at some point in the past, especially those arriving in Vienna, where 90% had previously participated in the labour market, compared to 84% in Kraków (S2 Table) (Chi^2^(1) = 11.18; p = 0.001). The majority had also been active in the labour market before leaving the country: in Kraków 63% of female respondents were (self-)employed back home, in Vienna 75% (Chi^2^(1) = 18.72; p<0.001). Previous self-employment is substantial, amounting to 13% in Kraków and 21% in Vienna (Chi^2^(1) = 14.79; p<0.001). Looking after home and family–before leaving Ukraine–was substantially more often reported among women in Kraków than in Vienna (11% versus 7%) (Chi^2^(1) = 7.29; p = 0.007).

Compared to labour market situation in their home country in 2021 (S3–S5 Tables), interviewed Ukrainian refugees are characterised by a higher share of employment and self-employment. The sample size in Vienna also allows for insights regarding male refugees, indicating an even larger proportion of self-employed among men arriving in Vienna (25%-30%; S2 Table).

The observation that roughly one out of two interviewed women worked full-time before leaving the country and that about 32% in Kraków and 20% in Vienna usually worked less than 20 hours a week and a further non-negligible group worked 20 to 34 hours per week (S6 Table), reflects care duties for minors. This is in line with the fact that the refugee population mainly consists of women with children. In the general working population in Ukraine, part-time work amounting to less than 20 hours per week was rare before the outbreak of the war (S5 Table). Correspondingly, (female) respondents in Vienna who intended to search for a job in Austria, often planned to work part-time (S6 Table). Regarding future plans, one out of four was still undecided at the time of the interview, and about 5% planned to continue education.

In the following, we discuss respondents employed before leaving Ukraine (including self-employment) focussing on occupational class structures using the International Standard Classification of Occupations (ISCO) and type of economic activity referring to the NACE classification (Nomenclature générale des activités économiques dans les Communautés Européennes). Even though item non-response was substantial (ISCO: 19%; NACE: 26%), available data still allow to discuss under- and overrepresentation of occupations and economic branches among Ukrainian respondents.

Findings regarding occupational status further confirm the high self-selection of Ukrainian respondents: More than 16% classify themselves as managers (ISCO 1), compared to 8% in Ukraine in 2021 (Table 1). In addition, a third of refugees report to be professionals (ISCO 2; 18% in Ukraine in 2021). Again, self-selection is more pronounced in Austria (19% managers and 36% professionals) than in Poland (10% and 28%, respectively). In contrast, the share of service and sale workers (ISCO 5) and workers in elementary occupations (ISCO 9) is higher in Poland than in Austria (7% versus 22%). Going into detail, many Ukrainian refugees have been educators and health professionals or, in particular in Austria, also administrative and commercial managers as well as legal, social and cultural professionals. Other more frequently reported occupations comprise general and keyboard clerks in Austria as well as personal and protective services workers and sales workers in Poland.

**Table 1:** Occupational status and type of economic activity.

	All			Women		
	Ukraine 2021	Kraków	Vienna	Ukraine 2021	Kraków	Vienna
**Occupational status**	%	%	%	%	%	%
ISCO 1: managers	8.1	9.6	18.7	7.0	9.2	18.5
ISCO 2: professionals	18.0	28.1	35.6	23.0	28.4	35.3
ISCO 3: technicians, associate professionals	11.8	7.5	7.2	15.9	7.5	7.6
ISCO 4, 6, 7, 8: clerical support/ skilled/ craft workers	28.1	15.8	12.1	14.4	16.3	12.3
ISCO 5, 9: service/sale workers, elementary occupations	33.9	21.6	7.2	39.7	22.0	7.1
No ISCO information available	---	17.5	19.2	---	16.7	19.2
Chi^2^(df)		56.81(5)	***		56.13(5)	***
**Type of economic activity**	%	%	%		%	%
NACE A, B, C, D, E, F: agriculture, manufacturing, etc.	36.5	8.6	10.1	25.7	8.2	9.0
NACE G, H, I: trade, transporting, accommodation, food services	31.1	17.5	10.5	29.4	17.7	10.3
NACE J: information and communication	1.9	4.1	7.6	1.3	3.6	7.1
Other service activities	---	---	---	43.6	---	---
NACE K: financial and insurance services	1.3	10.3	8.2	---	10.6	8.3
NACE L, M, N, O: administrative, professional, technical activities	11.9	8.2	9.2	---	8.5	9.3
NACE P: education	8.0	9.6	8.0	---	9.9	8.6
NACE Q: human health and social work activities.	5.9	6.5	7.7	---	6.7	8.0
Other types of economic activity	3.5	13.4	11.4	---	13.1	11.4
No NACE information available	---	21.9	27.3	---	21.6	28.2
Chi^2^(df)		18.07(8)	*		19.48(8)	*
N		292	780		282	702

Sources: State Statistics Service of Ukraine [51], UkrPL, and UkrAiA.

Note: Information on occupations for Ukraine 2021 is based on Labour Force Survey data; information on economic activity is based on data from Labour Force Survey, the state statistical observations over enterprises and administrative reporting. Data excludes occupied territories of the Autonomous Republic of Crimea, the city of Sevastopol, and a part of occupied territories in the Donetsk and Luhansk regions. Tests on differences between respondents from Kraków and Vienna

* p < 0.05

** p < 0.01

*** p < 0.001.

Although both the agricultural and the manufacturing sector represent a significant share in Ukraine’s economy (together 37% of total employment in 2021), only one out of ten refugees worked in these sectors (NACE A-F). Similar results are obtained for less knowledge-intensive services like trade, transportation or accommodation and food services (NACE G-I). Only 11% of Ukrainian refugees in Austria and 18% in Poland worked in these sectors, compared to 31% in Ukraine in 2021. Knowledge-intensive service activities are partly overrepresented among Ukrainian refugees. They often have work experience in financial and insurance services, education, human health and social work activities or, in Austria, also in the sector of information and communication.

### Choice of the host country

**Fig 3:**
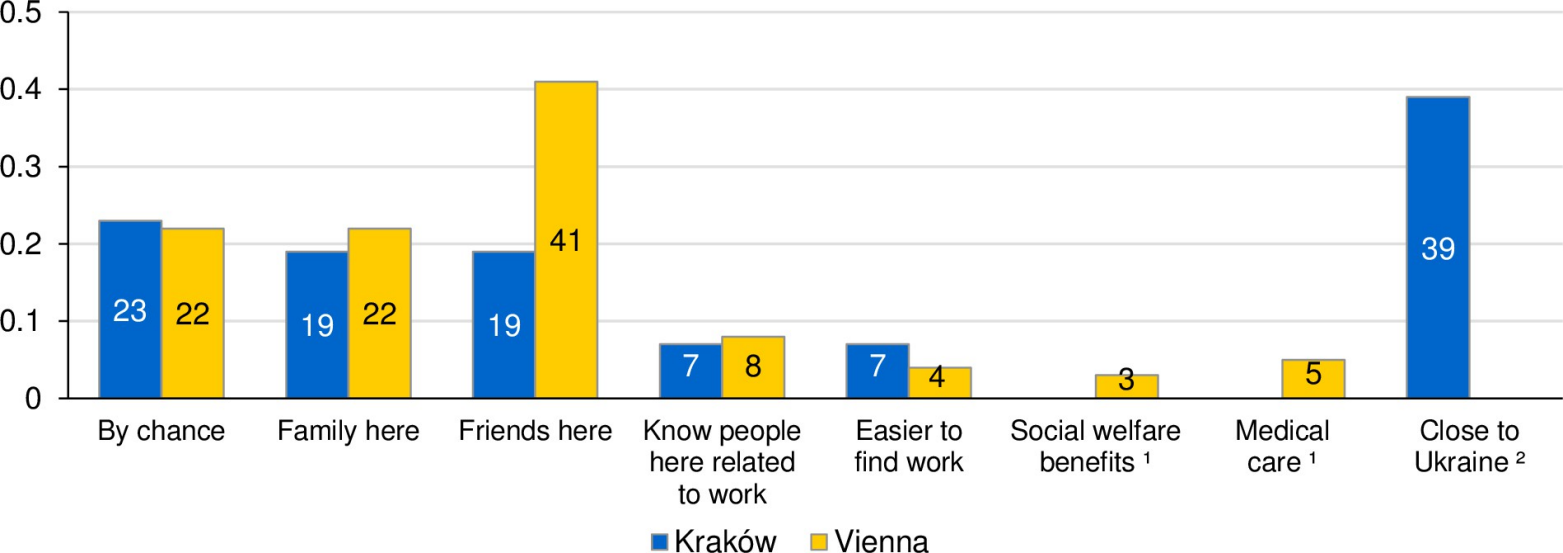
Reasons for choice of the host country, in %. Sources: UkrPL and UkrAiA. Note: ^1^ Option exclusive to Austrian survey; ^2^ Option exclusive to Polish survey. 95% confidence intervals for “By chance”: Kraków [19; 27]; Vienna [20; 25]; Chi^2^(1) = 0.06; p = 0.802; “Family here”: Kraków [16; 23]; Vienna [19; 24]; Chi^2^(1) = 1.40; p = 0.237; “Friends here”: Kraków [15; 22]; Vienna [39; 44]; Chi^2^(1) = 74.64; p<0.001; “Know people related to work”: Kraków [4; 9]; Vienna [7; 10]; Chi^2^(1) = 1.15; p = 0.285; “Easier to find work”: Kraków [5; 9]; Vienna [2; 5]; Chi^2^(1) = 8.83; p = 0.003.

Poland and Austria greatly differ when it comes to the pre-war Ukrainian diaspora size. In the case of Poland, ca. 1.3 million economic migrants to Ukraine have arrived in the country 2015–2021, and in Kraków alone the Ukrainian population at the end of 2021 was estimated between 70 and 80 thousand persons, which accounted for around 10% of the city’s entire population [64]. As for Austria, the pre-war population of Ukrainians was very small at about 12.7 thousand, with most of them living in Vienna [21]. These differences have had some implications for the decision of surveyed individuals when choosing the host country. For forced migrants from Ukraine who settled in Austria, the main reason for coming were the personal networks: having friends (41%), family members (22%) and knowing persons who enabled them to find jobs (8%, see Fig 3). In the case of Poland, the personal networks also played an important role, but a lower one than in Austria: 19% of respondents had family residing there prior to the war and additional 19% had friends staying in the country. Differences referring to having friends in the CoA are highly statistically significant (see note in Fig 3). Yet, the relevance of Ukrainian diaspora is visible in the response category “easier to find work”, which was selected by 7% of refugees in Poland compared to 4% in Austria. Many Ukrainian refugees might have known that Poland is a popular destination country for economic migration for their co-nationals, therefore it has a reputation of a relatively easy place to find employment [64]. But the most important reason to choose Poland was geographical proximity: 39% of surveyed refugees in Poland indicated this response. In fact, Kraków is directly connected with Lviv (major city in Western Ukraine) by train, while the A4 highway links Kraków with the Korczowa border crossing with Ukraine. This implies that within a 3.5 hours drive, a Ukrainian refugee located there can reach the border. Additionally, the presence of a large Ukrainian diaspora in the city, including Ukrainian cultural associations and businesses, such as community-run cafés, makes Kraków a place much closer to home in the socio-cultural sense.

The role of more generous social support in Austria than in Poland is visible in our surveys, but its effect is much lower than expected. Only 3% of respondents in Austria indicated that they chose their destination because of social welfare benefits, while additional 5% have asserted that the high level of medical care in Austria played a role in this aspect. Interestingly, both in Poland and Austria the share of respondents who have chosen the host country accidentally is similar: 23% for Kraków and 22% for Vienna.

### Return intentions

The Temporary Protection Directive of the EU allows Ukrainian refugees to live and work in EU member states. Therefore, those fleeing from the war in Ukraine have better opportunities to integrate into host societies than any other prior refugee group in Europe. Nevertheless, the crucial question whether they should go back to rebuild their country or not matters also to the people from Ukraine. As no one knows how the war will develop, reports of forced migrants about their inclination to stay or return are largely hypothetical. Still, the answers are important proxies indicating both refugees’ expectations and future developments for host countries.

**Fig 4:**
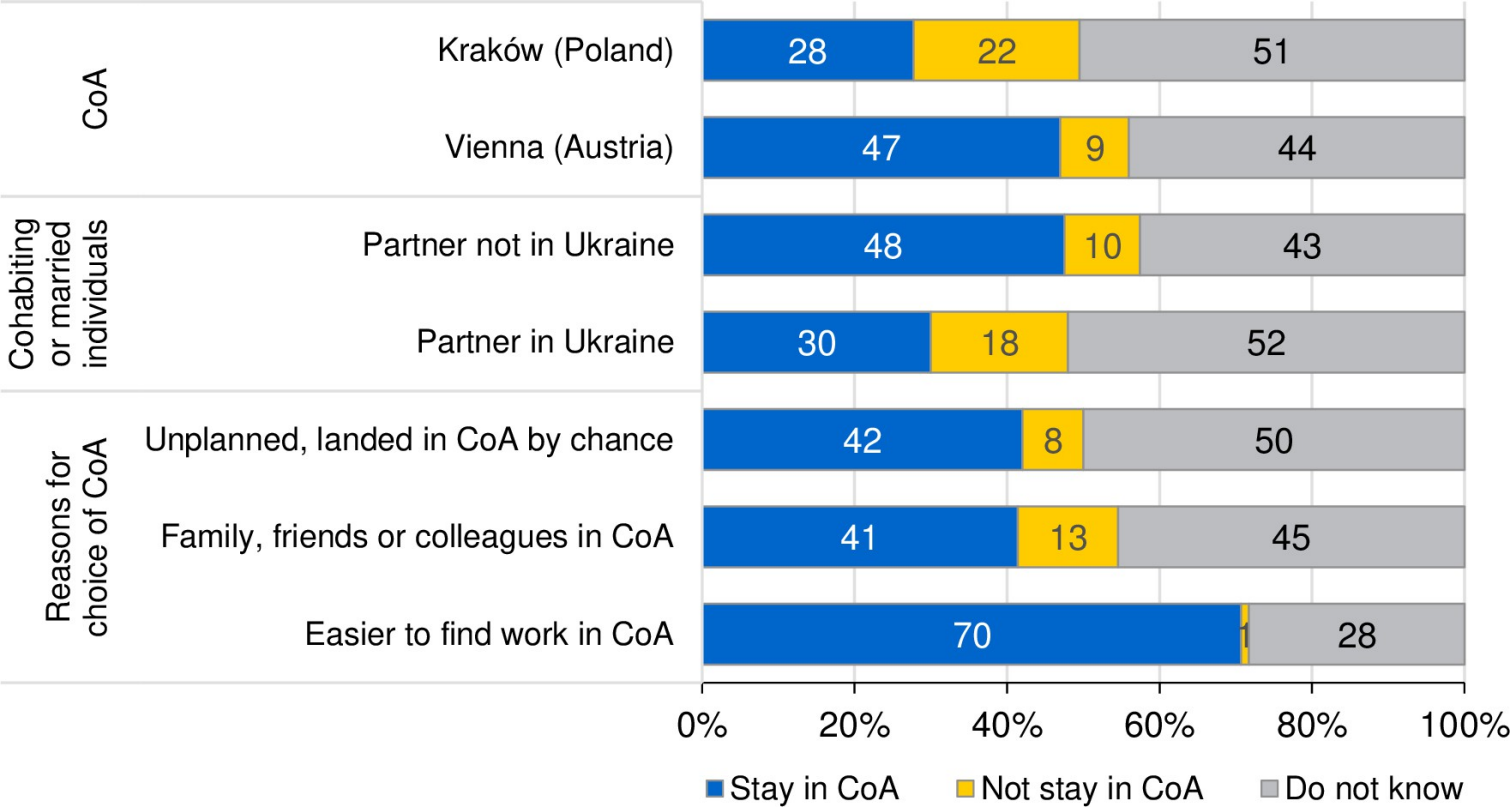
Intentions to stay in Country of Arrival (CoA), in %. Sources: UkrPL and UkrAiA. Note: 95% confidence intervals for “Stay in CoA”: Kraków [23; 32]; Vienna [44; 50]; Partner not in Ukraine [42; 53]; Partner in Ukraine [27; 34]; Landed in CoA by chance [37; 47]; Family, friends or colleagues in CoA [37; 47]; Easier to find work in CoA [60; 81]. See S7 Table for statistical tests.

Two questions were used to know more about this intention. The first one reads as follows: “Do you plan to stay in Poland/Austria?”. Answers most likely refer to the immediate future. The second question concerns long-term considerations about returning to Ukraine (e.g., “I want to return as soon as the war ends” or “I may return in case the war ends”). Both in Poland and Austria, a large part of respondents did not know whether they wanted to stay in the country of arrival or not (51% and 44%, respectively; Fig 4). The share of those wanting to stay, however, was larger in Austria (47%) than in Poland (28%). In Poland, even 14% indicated that they may return to Ukraine even if the war continues, compared to only 6% of respondents in Austria.

In a breakdown by gender, it is obvious that men more frequently wanted to stay than women, 16% stating that there is nothing to return to for them in Ukraine (S7 Table, including Chi^2^-tests). Regarding age, results are somewhat heterogenous. On the one hand, younger respondents (below age 25) reported more often that they want to stay. On the one hand, they also indicated more frequently that they want to return as soon as the war ends. By region, the statement that there is nothing to return was most often agreed to by respondents from Eastern Ukraine (17%) who also show the lowest share of surveyed refugees wanting to return to Ukraine as soon as the war ends (28%, compared to 42% among respondents from Kyiv) (S5 Fig and S7 Table). The situation is probably hardest in Eastern Ukraine and parts of Southern Ukraine which have been occupied by Russia. Return intentions are also lower among persons with at most secondary general education: Almost half of them want to permanently stay in the country of arrival, whereas less than a quarter want to return as soon as the war ends. Skills in the dominant language of the host country (i.e. German in Austria and Polish in Poland) are related to staying intentions. On the contrary, having a partner in Ukraine fosters return intentions. Finally, personal reasons for the choice of host country play also a role. On the one hand, our findings demonstrate that respondents who came to find a job overwhelmingly intend to stay (70%). On the other hand, refugees who arrived in the host country by chance are most often uncertain about staying or leaving (50% do not have any plan).

Multinomial logistic regression analyses confirm most messages of the bivariate results (Table 2, further information in S8 Table). However, descriptive results regarding age are not reflected in the regression models considering differences in socio-demographic characteristics and self-reported reasons for the choice of the host country. Pronounced differences are still observable between respondents in Austria and Poland and by varying personal reasons for the choice of the respective host country. In particular, those who chose a country of destination for employment reasons want to stay more often. The regression findings also suggest that the observed differences by destination can neither be explained by differences in socio-demographic characteristics of surveyed refugees nor by differences in the (asked) reasons for the host country.

**Table 2:** Estimated coefficients for intentions to stay in Country of Arrival (a) and to return to Ukraine (b), average marginal effects.

	(a) Do you plan to stay in Country of Arrival (CoA)?			(b) Which statements below may express your considerations about returning to Ukraine?				
	Stay in CoA	Not stay in CoA	Do not know	I have nothing to return to in Ukraine	I want to return as soon as the war ends	I may return in case the war ends	I may return even if the war continues	I do not have an idea, I do not know
**Survey**								
Kraków (Poland)	-.20***	.12***	.08*	-.01	.01	.06*	.07***	-.13***
Vienna (Austria), ref.	.00	.00	.00	.00	.00	.00	.00	.00
**Gender**								
Male, ref.	.00	.00	.00	.00	.00	.00	.00	.00
Female	-.04	-.03	.07	-.06**	-.07	.04	-.02	.10*
**Age**								
Below 25	-.01	.02	-.02	-.01	-.02	.02	.09***	-.09*
25+, ref.	.00	.00	.00	.00	.00	.00	.00	.00
**Residence before leaving Ukraine**								
Kyiv, ref.	.00	.00	.00	.00	.00	.00	.00	.00
Central Ukraine	.06	-.05	-.01	.06**	-.07	.00	.00	.01
Western Ukraine	.06	-.02	-.04	.01	-.15***	.04	.06*	.04
Southern Ukraine	.04	-.06*	.02	.02	-.05	.00	-.01	.04
Eastern Ukraine	.06	-.06*	.00	.12***	-.12***	.04	-.04*	.00
**Highest level of education**								
Secondary general education or less	.07	-.10***	.03	.04	-.08	.06	-.05**	.03
Vocational education	.00	.00	.00	.01	.01	.01	-.03	.01
Bachelor degree, ref.	.00	.00	.00	.00	.00	.00	.00	.00
Master degree or PhD	.00	-.03	.03	.03	.03	.02	-.01	-.08**
**Skills in dominant language of CoA**								
No skills in German/Polish language, ref.	.00	.00	.00	.00	.00	.00	.00	.00
Skills in German/Polish language	.10**	-.02	-.08*	-.01	-.04	.02	-.01	.05
**Relationship status**								
Cohabiting or married partner is not in Ukraine, ref.	.00	.00	.00	.00	.00	.00	.00	.00
Cohabiting or married partner is still in Ukraine	-.13***	.07**	.06	-.02	.15***	-.06	.05*	-.11***
Other family status: divorced, widowed, single (with or without partner)	.01	.00	-.01	.04	-.02	-.05	.01	.02
**Reasons for choice of CoA**								
Unplanned, landed in CoA by chance	-.02	-.09*	.11*	.04	-.02	-.02	-.01	.01
Reason “unplanned, …” not reported, ref.	.00	.00	.00	.00	.00	.00	.00	.00
Family, friends or colleagues in CoA	-.05	-.02	.07	.00	.03	-.02	.03	-.04
Reason “family or …” not reported, ref.	.00	.00	.00	.00	.00	.00	.00	.00
Easier to find work in CoA	.28***	-.23*	-.05	.04	-.09	.04	-.04	.05
Reason “easier to …” not reported, ref.	.00	.00	.00	.00	.00	.00	.00	.00
Other reason reported (geographical proximity, welfare/health system)	-.03	-.02	.06	.01	.07	-.09*	.04	-.02
No other reason reported, ref.	.00	.00	.00	.00	.00	.00	.00	.00
No reason reported	-.37*	.04	.34*	.08	.24	-.25	.09	-.15
At least one reason reported, ref.	.00	.00	.00	.00	.00	.00	.00	.00
**Cragg & Uhler’s R** ^**2**^		.19				.25		
**LL Intercept only**		-1,523				-2,250		
**LL Full model**		-1,387				-2,047		
**LR test**		272***				405***		
**AIC**		1.95				2.93		
**BIC**		-7,683				-5,704		
N		1,547				1,540		

Sources: UkrPL and UkrAiA.

Note: Models additionally control for survey mode (paper-and-pencil or web survey), religious denomination, subjective health, employment status before leaving Ukraine and parenthood status. Ref. = reference category.

* *p* < 0.05

** *p* < 0.01

*** *p* < 0.00

## Discussion and conclusion

Our results indicate high self-selection of surveyed Ukrainian refugees, in the key dimensions of formal educational attainment, socio-economic status and employment, as well as urbanity in Austria and, to a lesser degree, in Poland. While among the Ukrainian general population, 30% of persons aged 25–64 years hold a tertiary educational degree, this is the case for 66% in the Kraków sample and 83% in the Vienna sample. Similarly, we find higher employment rates in both surveyed refugee populations compared to the general employment rate in Ukraine. Notably, a third of respondents worked in academic professions. Previous self-employment is also high, which corresponds to observations among refugees in other contexts and suggests a pronounced entrepreneurial spirit among displaced persons [12, 65]. Lastly, 30% of respondents in the Viennese sample came from Kiev, which as the country’s capital had the highest GDP in Ukraine before the Russian invasion.

These indicators of high self-selection of refugees into European host countries have also been found by previous research [11, 12]. Our study thus contributes to the growing empirical evidence on consistent assortative patterns in forced migration. Such patterns are well-known and documented from labour migration and other less-coercive causes of migration [48, 62], as well as, in recent years, for the 2015/16 inflow of Middle-Eastern refugees into Europe due to, among other factors, high costs of irregular migration. Our findings on the socio-demographic composition of the Ukrainian displacement suggest that these regularities in patterns of self-selection also occur in forced migration contexts where legal routes to safety (visa-free entry into the EU for Ukrainian citizens) applied.

While strong in-group favouritism among military conflict victims, reliance on refugee cooperation [52, 54, 55] and dependence on financial and social resources are quite well documented in refugees’ self-selection into Europe, active choice of and familiarity with the host country are largely unique to Ukrainian displacement. Reasons for choosing Austria as a host country include, above all, strong social networks, but also high quality of life, well-functioning support structures, previous stays as tourists or students, as well as German language skills. This degree of familiarity is unusual in the context of European host countries, where previous cohorts of refugees from the Middle East or the Global South, while still self-selected in terms of education and social status [45], displayed much less knowledge and active choice of their host country. For policymakers and state agencies, host country choice and familiarity can become assets for facilitating integration. In contrast to Austria, however, the main factor for choosing Poland as host country was geographical proximity, which might point to a considerable share of surveyed arrivals only marginally embedded in diasporic networks. These individuals are most exposed to a risk of socio-economic marginalisation [66] and require special attention.

Building on these insights into refugees’ choice of host country, our findings may also reflect in which ways beneficiaries of temporary protection who reside in the immediate proximity of the Ukrainian border, namely in Poland, differ from those who went further away from Ukraine and reside in Western European countries, such as Austria. We tentatively conclude that the further Ukrainian refugees moved to the West, the more self-selected they tend to be and the lower their return intentions. Our data can be interpreted to reflect this pattern in the key dimensions of education, qualification and previous employment, language skills and social capital. Host countries should be aware of these specific characteristics of their refugee populations and adapt their integration policies accordingly [67], taking into account socioeconomic composition, intentions to return, and social ties.

For Western European host countries, the inflow of highly educated refugees means that rapid recognition of academic degrees or fast-track programs for qualification improvements [68] will be key for preventing stark mismatches between refugees’ qualifications and the jobs they end up working in. As de-qualification has been shown to be even more pronounced for women [69, 70], the predominantly female refugee population from Ukraine should be supported with free childcare, on-the-job training, and flexible working hours, including a continuation of pandemic-related remote work.

Finally, our results may also shed light on return and staying intentions of Ukrainian refugees and on the factors that may influence these. Overall, respondents based in Poland, from Kyiv, with lower language skills and those with a partner in Ukraine are more inclined to return and/or intend to return sooner, i.e. before the end of the war. As suggested by previous research on re-migration, women and children have a lower likelihood to return after forced migration [37, 71]. This applies in particular to protracted situations of war and displacement, when children become integrated in the host country’s schools and kindergartens and build a new social network. Uprooting them again from their (newly) familiar environment is a strategy that refugee families more often decide against compared to single refugees without family ties in the host country. While this overall pattern may apply to our study since women and children are overrepresented in both samples, a breakdown by gender shows that (the few) male respondents in our samples more frequently want to stay than women, with 16% of them stating that there is nothing to return to for them in Ukraine. This pattern could partly by explained by men’s eligibility for military service.

We also find that having a partner in Ukraine may both enhance the willingness to return and decrease the likelihood of staying in the destination country. In that sense, aspirations to reunite with one’s family may be identified as a factor in refugees’ re-migration patterns. From the perspective of countries of origin in a forced migration context, the policy of banning the emigration of (most) male individuals due to the military draft might act as an effective tool stimulating return migration of Ukrainian women when safety is restored. However, we cannot rule out the possibility of family reunification in the host country, i.e. male partners joining their families in Austria or Poland once the war has ended and consequently the emigration ban has been lifted.

Our findings can be interpreted to show that the willingness to stay in Kraków, a metropolis with an absorptive labour market and large Ukrainian diaspora, is significantly lower than the willingness to remain in Vienna–a much wealthier city, but with a relatively small Ukrainian population. We consider this relevant for further studies on refugees’ successful integration and adaptation to local conditions, as it may suggest that community support in the destination country must not be the decisive factor in shaping the decision to stay, to move on or to return. Rather, it seems that living and housing conditions, which, on average, are better in Vienna than in Kraków, may play a considerable role. In the former, the perspective of renting one’s own apartment with one’s salary and/or with state subsidies is higher, in particular since Ukrainian refugees in Austria receive those directly–in contrast to Poland, where it is private citizens hosting refugees who receive state funding for a limited number of days (mostly only 120 days). This finding has some important policy implications, as Austria could become an even more popular destination for Ukrainian refugees and migrants than before, given its growing diaspora and the relevance of social capital.

We identified a substantial share of respondents that asserted to have nothing to return to in Ukraine, most of whom stem from parts of Ukraine, which were heavily affected by the atrocities committed by the Russian troops and remain under Russia’s occupation. As the war and destruction continues, the share of individuals unwilling to return and/or even to cooperate with neighbours perceived as non-Ukrainians could unfortunately increase, with profound implications for inter-ethnic relations in Ukraine [56] as well as in the diaspora. This suggests that economic considerations are another relevant factor that may influence re-migration patterns, in particular as Ukrainians had in large numbers migrated to EU countries as labour migrants and seasonal workers before the outbreak of the war.

Our data has several limitations which need to be acknowledged. Both surveys follow a non-probability approach that does not allow the results to be generalised to the entire Ukrainian refugee population in Austria or Poland. Yet, the research designs carefully aim to minimise coverage error, in the absence of a sampling frame (e.g., access to national register data). For example, all refugees who wanted to stay in Vienna had to register at a unique first reception centre in Vienna. We assume that there is no major difference between the potential pool of Ukrainians arriving in Vienna and the target population at the above-mentioned reception centre, since appointments were randomly given to refugees. A comparison of the age and gender structure of the population surveyed in Vienna with data from the central population register in Austria on Ukrainians registered in Vienna by April 1^st^, 2022 confirms that, indicating no substantial bias in the Austrian sample. In Kraków, females are slightly over-represented as compared with the central population register in Poland (PESEL). Yet, when all the members of the household staying in Poland are considered, the data from our sample is fairly consistent with PESEL register. We do expect the choice of host cities, Vienna and Kraków, to lead to a sampling bias. While highly skilled refugees may be more inclined to stay in the country’s economically dominant city, the Polish region of Malopolska and in it, above all, Kraków were attracting large number of highly skilled migrants even before Russia’s invasion in Ukraine [72]. This pattern did not change during the inflow of refugees after February 24^th^. Hence, Kraków seems as good area to study the situation of refugees in Poland as any other big Polish city.

Finally, in terms of the sample size, the smaller sample in Poland compared to Austria introduces the possibility of greater biases in the Polish results. Additionally, differences in the self-reported network connections among respondents in the two countries stem from possible self-selection of less socially connected respondents in the Polish sample compared to the Austrian sample. These potential biases must be taken into account when interpreting the results, particularly regarding the selection of host country and further migration implications.

Overall, our findings on high self-selectivity together with high indecisiveness of surveyed refugees about their future return is alarming for Ukraine. Indeed, ongoing research points to declining return intentions of refugee since spring 2022 and rising intentions to stay in the host country [36], in particular in Western Europe, as our results suggest. Ukraine is thus faced with the growing threat of permanent brain drain, meaning loss of demographic, intellectual and socio-economic capital for the country, which might lead to significant (further) economic, cultural, and political damage. As a policy take-away, we recommend conceptualising the increasingly unlikely, immediate return of Ukrainian refugees to their homeland not as “brain drain”, but as “brain circulation”, with repeated stays for work or study in EU member states and (extensive) periods back in the home country, which will require human capital for rebuilding after the war. Hence, dual-intent support measures as recommended by the OECD may be key, fostering both integration in the host country and potential return to Ukraine [25]. Achieving this aim would require strong cooperation between countries and appropriate residence permits that legalise this form of circular migratory movement. Among others, Ukraine, Poland and Austria can achieve this common goal by developing cross-disciplinary research projects sharing state-of-the-art technologies and knowledge, which will strengthen human capital on both sides and encourage people to return, whether permanently or temporarily.

## Supporting information

S1 File UkrAiA-UkrPL survey. (DOCX)

S2 File UkrPL. English questionnaire.
(PDF)

S3 File UkrAiA. English questionnaire.
(PDF)

S1 Table Sample characteristics, in %. Sources: UkrPL and UkrAiA.
(PDF)

S2 Table Previous employment and planned labour market participation in host country, persons aged 18–59 years, in %. Sources: UkrPL and UkrAiA.
(PDF)

S3 Table Employment in Ukraine, 2021, in %. Sources: State Statistics Service of Ukraine [73].
(PDF)

S4 Table Employment status of persons aged 15–70 years, Ukraine, 2021, in %. Sources: State Statistics Service of Ukraine [73].
(PDF)

S5 Table Working hours of persons aged 15–70 years, Ukraine, 2021, in %. Sources: State Statistics Service of Ukraine [73].
(PDF)

S6 Table Usual working hours and plans for host country, persons aged 18–59 years, in %. Sources: UkrPL and UkrAiA.
(PDF)

S7 Table Intentions to stay in Country of Arrival (a) and to return to Ukraine (b), in %. Sources: UkrPL and UkrAiA.
(PDF)

S8 Table Confidence intervals of estimated coefficients (95%) for intentions to stay in Country of Arrival (a) and to return to Ukraine (b), average marginal effects shown in Table 2 in main text. Sources: UkrPL and UkrAiA.
(PDF)

S1 Fig Comparison of population in Ukraine and in EU-27 in 2021, by age and sex. Sources: Eurostat [74]. UNFPA [75]. Note: Full bars show the Ukrainian population, whereas empty bars show the total EU-27 (composition of 2021) population.
(TIF)

S2 FigDemographic structure of surveyed refugee populations in Kraków and Vienna.Sources: UkrPL and UkrAiA. Note: Figures include respondents as well as their children and partners if they stayed with them in Poland (N = 927) or Austria (N = 2,194), respectively.(TIF)

S3 Fig Allocation of respondents, GDP per capita, and population in Ukraine. Sources: UkrPL and UkrAiA. UNFPA [52].
(TIF)

S4 Fig Correlation between respondents’ origin and GDP (left) as well as rate of students (right). Sources: UkrPL, UkrAiA and State Statistics Service of Ukraine [55]. Note: The rate of respondents in the population is calculated by dividing the number of respondents in the UkrPL/UkrAiA-survey originating from the region by the number of persons living in the region in 2021, multiplied by 100. The rate of students is calculated by dividing the number of students in the region by the number of persons living in the region in 2021, multiplied by 100.
(TIF)

S5 Fig Intentions to return to Ukraine, in %. Sources: **UkrPL and UkrAiA. See also S7 Table.**
(TIF)
