# Supplementary material for: High self-selection of Ukrainian refugees into Europe: Evidence from Kraków and Vienna
Source: PLoS One. 2023 Dec 20;18(12):e0279783. doi: 10.1371/journal.pone.0279783 (PMC10732457; doi:10.1371/journal.pone.0279783)
Supplement: S1 File — (DOCX) [file pone.0279783.s001.docx]

S1 File: UkrAiA-UkrPL Survey

The research projects *Ukrainian Arrivals in Austria* (UkrAiA) and *Ukrainian Arrivals in Poland (UkrPL)* aimed to shed light on Ukrainian displaced persons in Austria and Poland who left their homes due to the Russian war of aggression. These two projects sought to establish an evidence base for understanding the needs and resources of displaced individuals in the areas of integration, education, labour market and housing. They were led by researchers from the Vienna University of Economics and Business (WU) and the Austrian Academy of Sciences (OeAW) in Austria and from the Multiculturalism and Migration Observatory (MMO), as well as the Centre for Advanced Studies of Population and Religion (CASPAR) at the Cracow University of Economics in Poland.

The UkrAiA survey was a rapid-response survey and provided the first reliable data on Ukrainian displaced persons in Austria. The field phase took place between April and June 2022, during the early stages of the war. Data collection was carried out using a multi-mode approach (PAPI and CAWI) following convenience sampling. The final sample consisted of $N$=1,094 Ukrainian individuals aged 18 and above. The survey design was approved by the ethics committee of the Vienna University of Economics and Business and follows the university’s as well as international refugee studies’ ethical guidelines [1, 2]. During the field phase of the survey financial support was provided by the City of Vienna and the Vienna Social Fund. Furthermore, the University of Applied Sciences Salzburg supported the CAWI design.

Similar to Austria, the field phase of the UkrPL survey in Poland took place between May and June 2022. The final sample consisted of $N$=472 Ukrainian individuals aged 18 and above. In addition to the items included in the UkrAiA survey, the UkrPL survey also incorporated a set of questions assessing refugees’ perception of various elements of the reception system as well as actors involved in the support of Ukrainian refugees. The survey design was approved by the ethics committee of the Cracow University of Economics and the field phase of the survey was supported financially by the Cracow University of Economics and the Multiculturalism and Migration Observatory.

## 1. Survey Design

Both surveys collected individual-level data on Ukrainian displaced persons. Since they were designed as household surveys, they provided additional information on potential partners and children. The survey items were based on existing survey instruments, such as the Labour Force Survey, the World Value Survey, the Survey of Health, Ageing and Retirement in Europe, the Generations and Gender Survey, a social survey among Syrian and Afghan refugees arriving in Austria in 2015 [3] as well as a survey on the integration of immigrants in Kraków and Malopolska Region [4] covering the following areas:

- **Socio-demographic characteristics:** age, gender, origin, citizenship, religion, social class and income group
- **Human capital and qualifications:** language skills as well as educational, vocational, and academic qualifications
- **Employment:** labour market experience before the flight and intentions for future work
- **Family context:** marital status as well as family constellation before and after flight
- **Health:** physical health and impairment to work
- **Social capital:** support and social networks
- **Housing:** living situation before and after flight
- **Circumstances of flight:** costs, constellation and timeline of flight
- **Intentions for the future:** family reunification, remigration
- **Attitudes and values:** gender roles, democracy and confidence in international institutions (only in UkrAiA)
- **Reception system:** evaluation of various elements of the reception system and actors involved in provision of support to Ukrainian refugees (only in UkrPL)

The surveys were prepared in Ukrainian, Russian and English language, including pre-tests on cultural and linguistic particularities [5, 6]. For example, some topics may have been perceived as taboo (e.g., a third gender option) or may have only worked with specific country context information (e.g., the exact wording of educational paths in the given country). The survey items were adopted in an iterative process in collaboration with interpreters possessing in-depth linguistic and cultural knowledge. Additional pre-tests were conducted in the field. No further controversial, difficult to understand, misinterpreted or sensitive survey items were identified.

## 2. Data Collection

All personal data was anonymised and processed over secure connections. Furthermore, data access was monitored and recorded by the research team. The servers for data entry and processing were located in Austria.

### Vienna

The field phase of the UkrAiA survey took place between April 11 and June 8, 2022. No available sampling frame existed (e.g., access to national register data) that would have allowed us to accurately determine the probability of Ukrainian arrivals being selected for our sample. Thus, we followed convenience sampling, a non-probability approach that does not allow the results to be generalised across the entire Ukrainian refugee population in Vienna. However, the particularity of the location in which the fieldwork took place allowed us to largely minimise the possibility of a coverage error bias.

The fieldwork was conducted at a unique location for Ukrainian refugees arriving in Vienna. The first reception centre for displaced people from Ukraine was located at the Austria Center Vienna. There, Ukrainians had to register with the police (if they had not already done so), apply for primary care and housing assistance. In addition, psychosocial emergency support, ad-hoc sleeping places, medical care, food, sanitary items as well as COVID-19 tests were provided. The most important peculiarity of our sample is that this reception centre was the only one in Vienna during the time of data collection. Thus, all displaced Ukrainians who intended to stay in Vienna had to go there, i.e. not those Ukrainians who were only transiting. Thus, major differences between the potential pool of all Ukrainian arrivals in Vienna and the target population at the reception centre are unlikely, since appointments there were given to refugees in no particular order. A comparison of the demographic structure in our sample with data from the central population register in Austria (ZMR) confirms this assumption. (see section 4 “Sample” of this document).

During the field phase, a multi-modal approach was applied: paper and pencil interviews (PAPI) and computer-assisted web interviews (CAWI). For the latter, we created individual QR codes leading to the web-survey, which could only be used once. Hence, multiple answers from the same person or several persons of one household were prevented, as well as uncontrolled distribution or unintentional snowballing. Our reasoning behind using both survey methods was to provide more flexibility. First, a priori field observations showed that conditions in the field varied greatly from day to day, from short waiting times with sufficient seating available up to hours of standing in queues. Thus, we provided the option to fill out the questionnaire on cell phones, tablets or laptops (a free internet connection was provided on-site). Second, parts of the refugee population included non-digital natives like some older respondents or persons reluctant to use their mobile phones for longer periods of time - as well as persons unwilling to fill out the CAWI for other reasons. This way, we aimed to reduce potential bias resulting from not capturing persons with low education, high age, etc. by providing a paper and pencil version as well.

### Kraków

The field phase of the UkrPL survey took place between May 5 and June 15, 2022. The data collection in Kraków also followed convenience sampling due to the lack of precise information about the analysed population. The survey was carried out in the form of paper and pencil interviews (PAPI) by trained Ukrainian researchers in numerous locations where Ukrainian refugees gathered. The research team made sure that the places chosen covered diverse groups of Ukrainians arriving in Poland after 24^th^ February. The main locations where data collection took place were the Tauron Arena (the personal registration (PESEL) and UNHCR cash benefit registration spots), where 34% of the respondents were approached by the survey team, and support centres (a warehouse with food and medicine and a consultation centre for refugees). Other important places were the Galeria Plaza where the Szafa Dobra (a distribution centre of free clothes for refugees) and one of the collective shelters were situated. Further locations included the Ukrainian Consulate in Kraków, the World Kitchen on the Main Railway Station and other public spaces in Kraków.

## 3. Recruitment and Training of Interviewers

### Vienna

The data collection was conducted by a team of interpreters and students under scientific supervision. Members of the scientific team oversaw the entire data collection process and were responsible for handling any difficult or unexpected situations. Student helpers and interpreters were recruited from different universities and trained to minimise data collection errors. The interpreters were fluent in Ukrainian and/or Russian (in addition to German and English language skills) and were assisted by German and English-speaking student helpers. At the fieldwork location, multiple humanitarian organisations were on-site for any kind of ad-hoc support (e.g., medical personnel, police, or psychological counselling). To prevent re-traumatisation of the displaced individuals [7], students and interpreters were instructed to not interfere in unforeseen situations. In such situations, the academic team was informed immediately and coordinated with the professional colleagues on site to provide help. Field observations were monitored in detail. After every day of fieldwork, student helpers, interpreters and academic supervisors filled out an observation diary. At the end of the field phase, psychological counselling was offered to student helpers and interpreters, to give them an opportunity to process their experiences.

### Kraków

The research team in Kraków consisted of senior researchers and research assistants. One senior researcher and four research assistants were refugees themselves, which proved very important during the process of planning, designing the questionnaire and data collection. For instance, they had access to areas restricted to refugees and thus were able to conduct interviews there. During data collection, the scientific team oversaw the process and was in regular contact with the research assistants to immediately discuss any issues emerging. One member of the scientific team who also participated in the data collection regularly discussed field observations with the research assistants.

## 4. Sample

During both field phases, 2,528 persons agreed to participate in the surveys and were given a paper questionnaire ($S^{PAPI}$=2,120). In addition, in Austria, some persons received an information sheet with a one-time QR code that led to the online version of the questionnaire ($S^{CAWI}$=408). A total of 1,736 interviews were completed in Austria and Poland. Due to data inconsistencies, some observations were excluded in both surveys (see information on internal validation). Thus, the final sample consisted of $N$=1,572 persons ($N^{AUS}$=1,094, $N^{POL}$=472). The vast majority of respondents were women, though the share of men in the Austrian sample was higher (10.5%) than in the Polish sample (3%). The mean age of men was only slightly higher (Austria: 39.8 years, Poland: 40.4 years) than that of women (Austria: 38.9 years, Poland 39.2 years).

Figure 1 shows the population size in 2021 and region of origin of respondents. Map colours indicate the population in 2021, yellow circles show the share of respondents from that region in the Austrian sample, and red circles indicate shares in the Polish sample. In both surveys, most respondents came from densely populated regions. The major difference is that the relative number of respondents from Kyiv is considerably higher in the Austrian sample than in the Polish one.

**S1 File Figure 1. Population size in 2021 and region (oblast) of origin of respondents.**

**
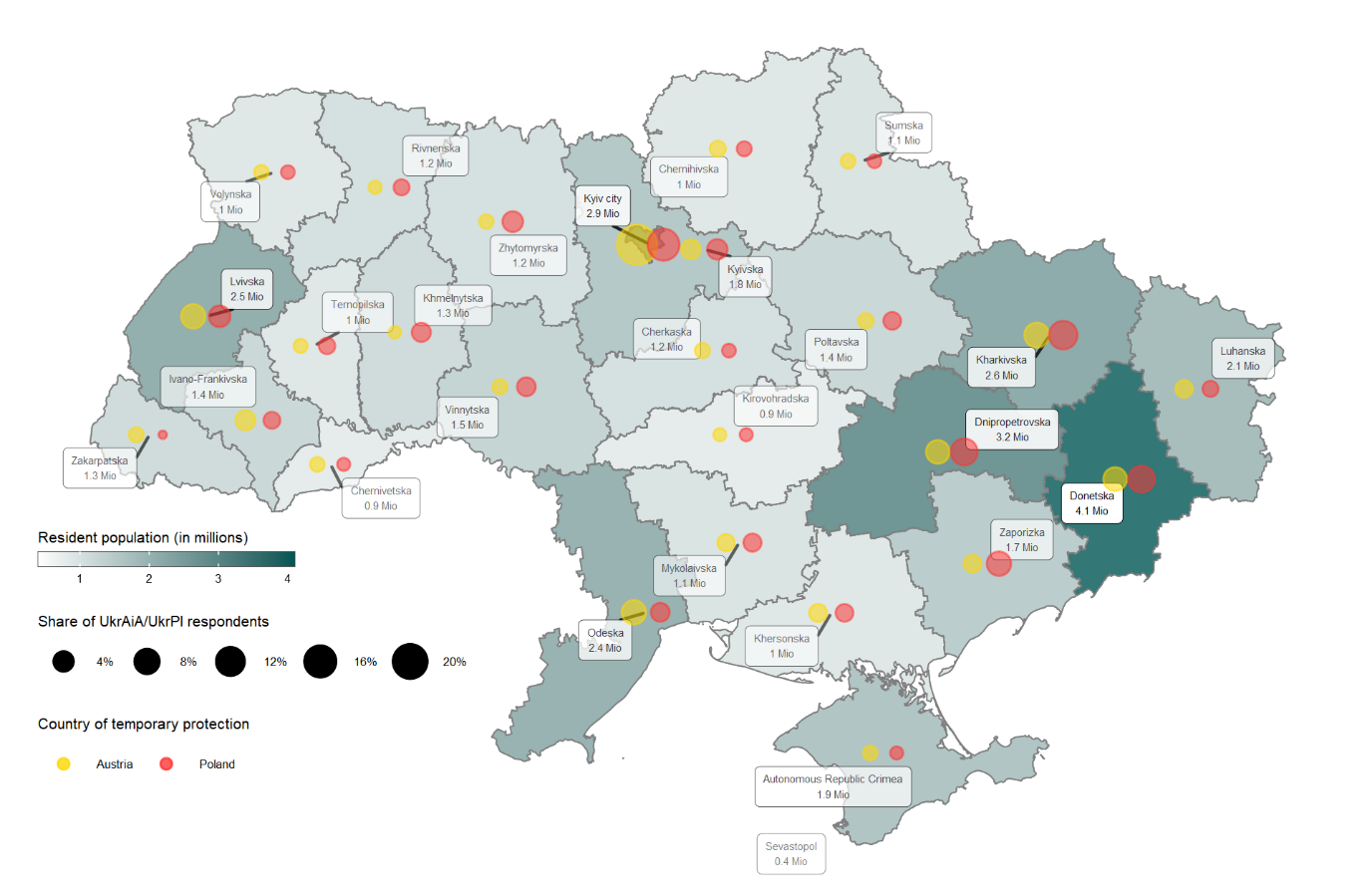
***Sources:* UkrAiA, UkrPL, State Statistics Service of Ukraine [8].

*Note:* Map colours show the population size (in millions) per region in 2021. Circle colour indicates the country of temporary protection while circle diameters indicate the relative contribution of cases to the total sample (per country of temporary protection).

The questionnaires included selected demographic characteristics of the nuclear family. Thus, data comprise sex, age and whereabouts at the time of the interview of partners/spouses (Austria: 661; Poland 272 persons) and children (Austria: 1037, Poland: 509 persons). In addition, the Austrian survey gathered information about the education and labour market experience of the partners/spouses.

### External Validation

#### Austria

Data on the whereabouts of the nuclear family allowed us to compare the sampled population with Ukrainians registered in the country/city of arrival with regard to sex and age (Figures 2-6). The survey conducted in Poland was carried out in Kraków, which is not the country’s capital as is Vienna, but nevertheless the second most populous city in Poland. It is also one of the major cities in Poland which had already hosted a sizeable Ukrainian population before the war [9], which again significantly grew after 24^th^ February 2022. The situation in Poland also differs from Austria that Ukrainian refugees are hosted all across the country and not concentrated in specific cities like Vienna [10].

**S1 File Figure 2. Ukrainians in Vienna.**

| Persons surveyed in UkrAiA | Persons registered in Vienna by April 2022 |
| --- | --- |
| 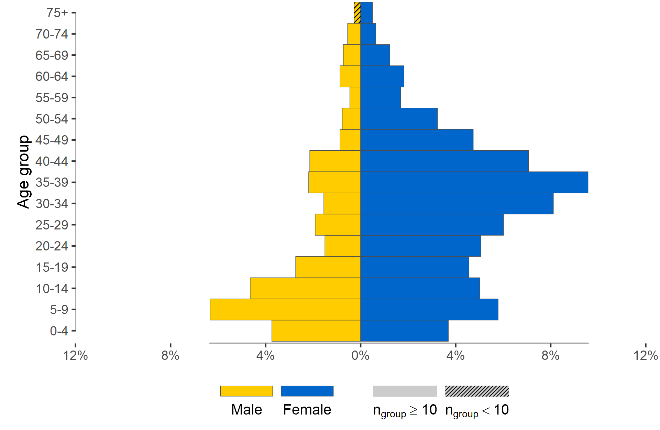 | 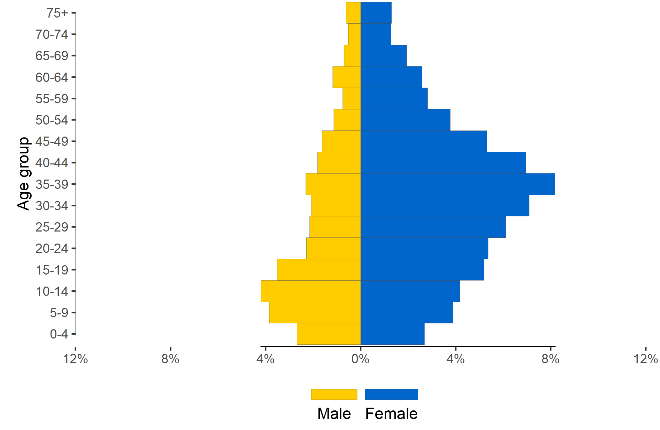 |

*Sources:* UkrAiA, Statistics Austria [11], additional data on Ukrainians provided upon request by Statistics Austria.

*Note:* The age pyramid on the left includes 2,194 persons (1,094 respondents, 249 partners/spouses, 738 minor children and 113 adult children) living in Vienna by the time of the interview. The age pyramid on the right includes 22,277 Ukrainians and registered in Vienna on April 1, 2022.

For Austria, Figure 2 visualises the age-sex distribution of persons surveyed in the UkrAiA survey compared with Ukrainians registered in Vienna by April 2022, as only quarterly data is available. We can observe a rather similar distribution across both population pyramids. Young adults aged 19-29 as well as older persons over the age of 60 years seem somewhat underrepresented, while children (aged 0-18) tend to be overrepresented in the UkrAiA sample. Considering that the target population are refugees who arrived in Austria since the beginning of the war, we are able to make a crude estimate of this group by subtracting Ukrainians registered by January 1, 2022 from those registered by April 1, 2022 (right panel of Figure 3). This way we find an even better fit in the distribution of the surveyed UkrAiA refugee population in comparison to the “newly arrived” Ukrainians in Vienna. The remaining notable differences are a minor underrepresentation of male teenagers and a minor overrepresentation of men in middle adulthood. According to the ex-post validation of our sample, we can conclude that the UkrAiA sample is quite representative the overall Ukrainian refugee population in Vienna in terms of age and sex structure.

**S1 File Figure 3. “Newly arrived” Ukrainians in Vienna in 2022.**

| Persons surveyed in UkrAiA | Persons registered in Vienna |
| --- | --- |
| 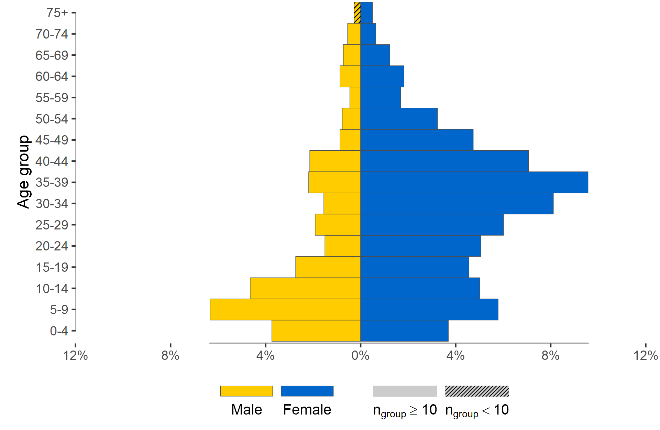 | 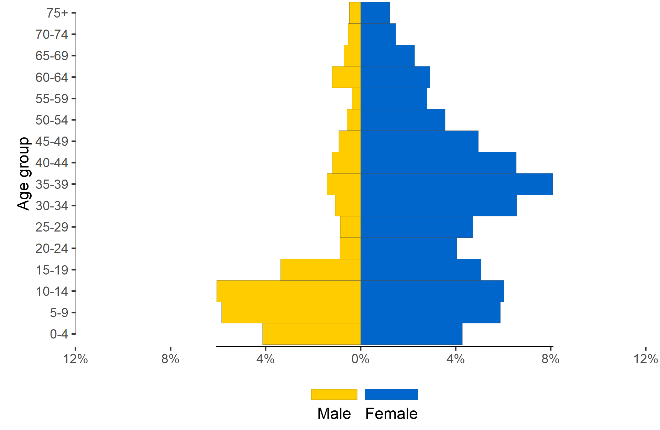 |

*Sources:* UkrAiA, Statistics Austria [11], additional data on Ukrainians provided upon request by Statistics Austria.

*Note:* The age pyramid on the left includes 2,194 persons (1,094 respondents, 249 partners/spouses, 738 minor children and 113 adult children) all living in Vienna by the time of the interview. The age pyramid on the right includes 13,323 Ukrainians registered in Vienna who likely arrived after January 1, 2022.

One peculiarity of the Austrian situation is that the city of Vienna is by far the largest city in Austria, which, with 1.93 million inhabitants, has over six and a half times more inhabitants than the second largest (Graz) with only 0.29 million. Almost a fourth of the Austrian population lives in the city of Vienna proper and approximately a third of the Austria population lives in the metropolitan area of Vienna. Furthermore, the share of refugees in Austria living in Vienna has been even higher in the last decade, with almost half of the Syrian and of the Afghan populations (47% and 47%) living there [12]. Recent data from April 2022 indicates that the situation with regard to Ukrainian refugees in Austria is quite similar, with 41% of that group living in the city of Vienna [11] (additional data on Ukrainians in Vienna and the remaining part of Austria in April 2022 was provided upon request by Statistics Austria).

**S1 File Figure 4. “Newly arrived” Ukrainians in Austria in 2022.**

| Persons surveyed in UkrAiA | Persons registered in Austria |
| --- | --- |
| 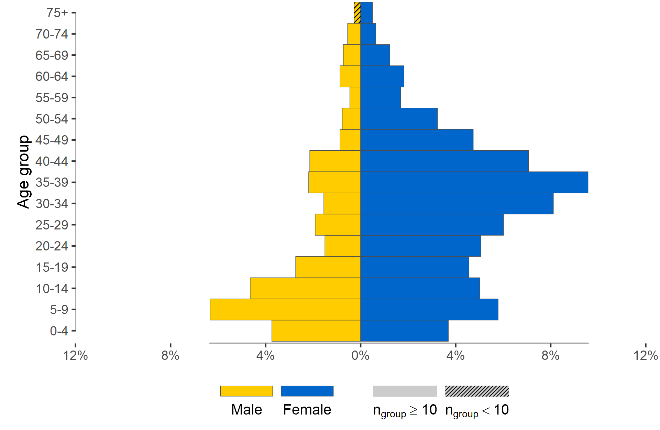 | 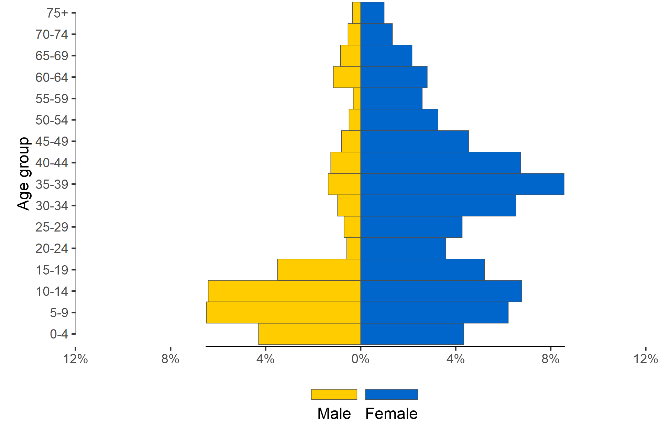 |

*Sources:* UkrAiA, Statistics Austria [11], additional data on Ukrainians provided upon request by Statistics Austria.

*Note:* The age pyramid on the left includes 2,194 persons (1,094 respondents, 249 partners/spouses, 738 minor children and 113 adult children) living in Vienna at the time of the interview. The age pyramid on the right includes 38,547 Ukrainians registered on April 1, 2022, who likely arrived in Austria after January 1, 2022.

Figure 4 shows a comparison of the age-sex profile of the sample with Ukrainians registered in Austria on the first of April 2022 again subtracting Ukrainians already registered on January 1, 2022 (Figure 4). The distribution is very similar to that of Ukrainians in Vienna, partially because Ukrainians in Vienna make up a big part of all Ukrainians in Austria as indicated before. Nevertheless, a direct comparison between the age-sex distribution of Ukrainians in Vienna and the Austria without Vienna shows that they are equally similar, despite the very special situation of Vienna. This leads to the tentative conclusion that the age-sex distribution of the UkrAiA sample not only allows valuable insights on displaced persons originating from Ukraine living in Vienna, but also those staying in other parts of Austria.

#### Poland

Figure 5 provides a comparison for the Polish case. Similarly, we compare persons surveyed in UkrPL with Ukrainians registered in Kraków who received a PESEL (Universal Electronic System for Registration of the Population) number as of April 13, 2022 [13]. The PESEL number is a personal identification number for all persons living in Poland permanently or temporary for more than 2 months, including persons without Polish citizenship. Since 16^th^, March, 2022 Ukrainian arrivals in Poland could register and apply for a PESEL number even without having been in Poland for more than two months. Due to this change, not every Ukrainian arrival had been registered by April/May 2022, though the vast majority had already applied for a PESEL number.

**S1 File Figure 5. Ukrainians in Kraków.**

| Persons surveyed in UkrPL | Registered refugees in Kraków |
| --- | --- |
| 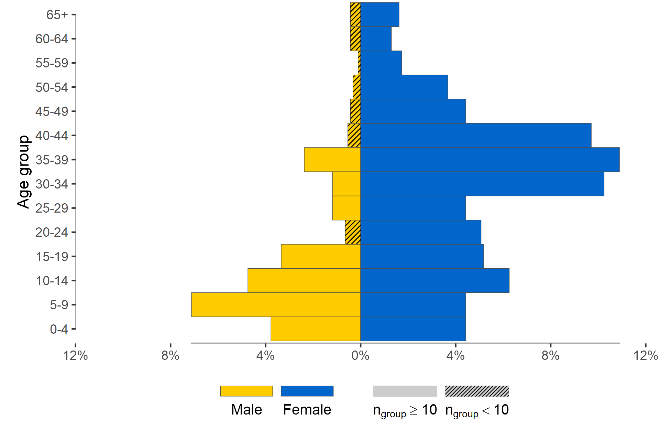 | 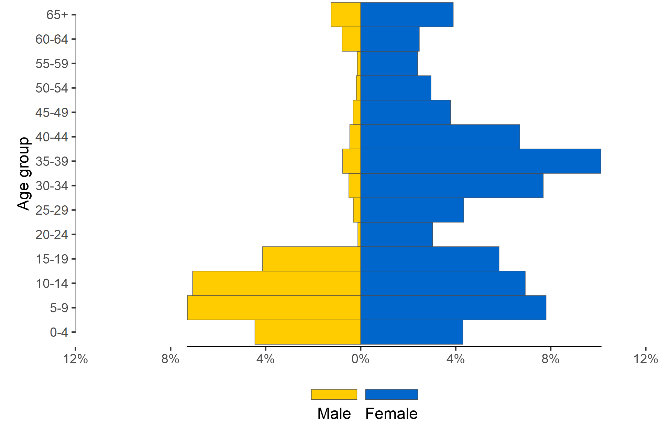 |

*Sources:* UkrPL, Poland’s Data Portal [13].

*Note:* The age pyramid on the left includes 927 persons (472 respondents, 55 partners/spouses, 339 minor children and 61 adult children) all living in Kraków by the time of the interview. The age pyramid on the right includes 12,670 Ukrainians registered as living in Kraków on April 13, 2022. Hatched bars on the left-hand side indicate low sample size (below 10 persons) in that age group.

The survey sample underrepresents teenagers, while slightly overrepresenting women aged 20-24, 30-34 and 40-44. Given the low number of men in the sample, we cannot draw any robust conclusions on the spike of men in the age group 35-39. Nevertheless, we acknowledge that the age-sex distributions of the Polish survey widely overlap. Furthermore, it resembles that of Ukrainians registered for temporary protection by mid-April in all of Poland relatively well (see Figure 6). Due to data availability issues, we cannot derive even a rough estimate for the group of the “newly arrived” Ukrainians in Kraków and compare it with the UkrPL sample.

**S1 File Figure 6. Ukrainians in Poland.**

| Persons surveyed in UkrPL | Registered refugees in Poland |
| --- | --- |
| 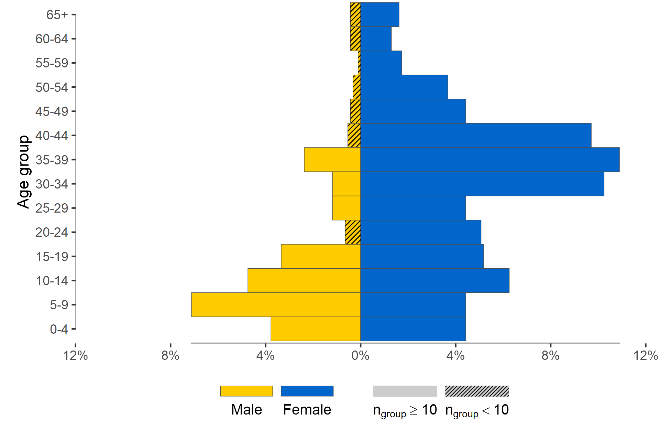 | 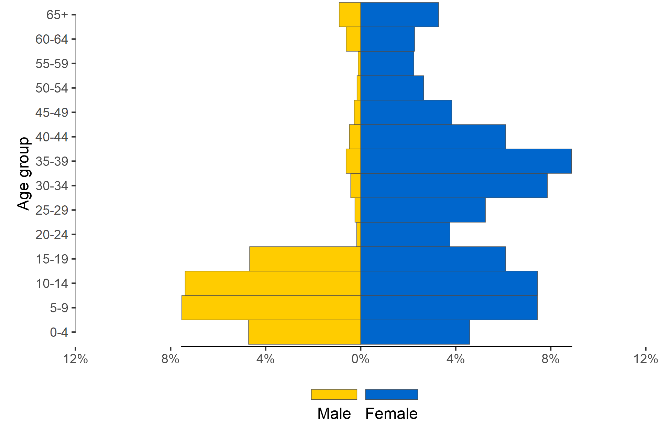 |

**Sources:** UkrPL, Poland’s Data Portal [13].

**Note:** The age pyramid on the left includes 927 persons (472 respondents, 55 partners/spouses, 339 minor children and 61 adult children) all living in Kraków by the time of the interview. The age pyramid on the right includes 907,856 Ukrainians registered as living in Poland on April 13, 2022. Hatched bars on the left-hand side indicate low sample size (below 10 persons) in that age group.

### Internal Validation

Cases with missing information on age and/or sex were excluded from the sample. In the Austrian case, due to the sufficiently big gross sample size, cases with missing answers to further key questions such as citizenship, country of birth and date of arrival in the country of temporary protection were excluded as the eligibility could not be determined. This reduced the sample from 1,901 questionnaires, in which at least one question was answered, by 156 cases to 1,745 cases. Of these, 24 were ineligible as they did not adhere to our sampling criteria, for example arriving in the country of temporary protection before the beginning of the war, not being of Ukrainian citizenship or being minor (below the age of 18). Non-submitted CAWI questionnaires are by definition aborted, whereas we defined those PAPI questionnaires as aborted, where the last regular question Q67 (a question on relative income in the home country) and all following questions Q68 (“We now have come to the end of our interview…”) and Q69-Q70 (questions for contact details for future interviews) had all been left unanswered. We excluded 115 PAPI interviews as aborted by this definition as well as 13 started, but unsubmitted CAWI interviews. Furthermore, we excluded 21 partial interviews which were missing important explanatory variables (education, family status and region of origin). The final sample thus consisted of 1,572 cases.

#### Response and cooperation rate

The response rates $RR_{4}$ and $RR_{6}$ [14] as defined by AAPOR assume that the eligibility of sample units is mostly known before conducting an interview. Refusals are defined to be part of the group of sample units with final disposition state ($R$), i.e. are eligible units that either refused to participate or stopped the interview. In both refugee populations, Vienna and Kraków, no lists of potential respondents let alone their demographic properties were available. Thus, eligibility/ineligibility due to units being out of sample could not be determined ex-ante. According to the AAPOR Standard definitions, all immediate refusals to participate as well as unreturned questionnaires would both have to be classified as $UO$ (Unknown Eligibility – Other reasons). To better discern them, we split the final disposition into three groups: $UR$, $UO’$ and $UO’’$ (see Table 1). Furthermore, we categorised incomplete/aborted cases where eligibility could be positively determined as $R$ (Eligible - Break-off).

As interviews were conducted only in centres installed to process Ukrainian arrivals, the estimated eligibility was expectably high with $e_{AT}$=0.986 and $e_{POL}$=0.998. To calculate the response rates, we replaced $UO$ with $(UR+UO’+UO’’)$ in the AAPOR formula for the lower and upper bounds of the response rate ($RR_{4}$ and $RR_{6}$):

$$RR_{4}=\frac{I+P}{\left( I+P \right)+\left( R+NC+O \right)+e(UH+UR+UO’+UO’’)}$$

$$RR_{6}=\frac{I+P}{\left( I+P \right)+\left( NC+O+R \right)}$$

The upper bounds ($RR_{6}$) for these response rates are incorrectly high, due to the impossibility to determine eligibility ex-ante conducting an interview. Thus, refusals and a considerable number of completed interviews end up in the group of unknown eligibility. To account for this, we additionally calculated an adjusted upper bound of the response rate ($RR_{6}^{'}$). It includes those cases that refused to participate before eligibility could be determined ($UR$), weigh it with the estimated eligibility $(e$) and add them to the denominator.

$$RR_{6}^{'}=\frac{I+P}{\left( I+P \right)+\left( NC+O+R \right)+eUR}$$

For UkrAiA, these adjusted upper bound and adjusted average response rate are much lower ($RR_{4-6}^{AT^{'}}$=0.58) than the unadjusted average response rate ($RR_{4-6}^{AT}$=0.73).

We then calculated cooperation rates with the formula:

$$COOP_{1}=\frac{I}{\left( I+P \right)+(R+O)}$$

Computing cooperation rates using the regular formula does not yield meaningful values either. Thus, we analogously calculated an adjusted cooperation rate, which we define as:

$$COOP_{1}^{'}=\frac{I}{\left( I+P \right)+\left( R+O \right)+eUR}$$

For the adjusted cooperation rate, we still find a relatively high rate of $COOP_{1}^{AT^{'}}$=0.59. This is considerably lower than those calculated for surveys conducted among refugees in Austria who had arrived during and following the refugee waves of 2015. Kohlenberger et al. [15] report similar adjusted cooperation rates of 0.81, though they describe a guided CATI survey among refugees who had been in Austria for years. Thus, it was to be expected that cooperation rates of Ukrainians filling out paper questionnaires on the spot while waiting for appointments would yield lower rates.

**S1 File Table 1. Final disposition and response rates.**

|  | | **Country of temporary protection** | | | **AAPOR (Adapted)** | **Interview started** |
| --- | --- | --- | --- | --- | --- | --- |
|  |  | **Total** | **Austria** | **Poland** |  |  |
| **Sample** | Sample Units PAPI | 2,120 | 1,620 | 500 |  |  |
|  | Sample Units CAWI | 408 | 408 | - |  |  |
|  | **Sample Units Total** | **2,528** | **2,028** | **500** |  |  |
| **Unknown Eligibility** | Unknown Eligibility – Non-Contact | - | - | - | $UH$ | No |
|  | Unknown Eligibility - Other (Refusal / PAPI questionnaires not returned / CAWI questionnaires not started) | 627 | 627 | - | $UR$ |  |
|  | Unknown Eligibility - Other - Interviews missing answers to key demographic variables (sex, age) | 80 | 59 | 21 | $UO^{'}$ | Yes |
|  | Unknown Eligibility - Other - Interviews missing answers with further key variables (citizenship, country of birth, date of arrival in host country) | 76 | 76 | - | $UO''$ | 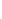Yes |
| **Ineligible** | Ineligible - Out of sample (date of arrival in host country (before February 2022, age (below 18), citizenship) | 24 | 23 | 71 |  | Yes |
| **Eligible** | Eligible - Non-contact | - | - | - | $NC$ | No |
|  | Eligible - Other | - | - | - | $O$ |  |
|  | Eligible - Response - Break-off (aborted interviews) | 128 | 128 | - | $R$ | Yes |
|  | Eligible - Response - Partial Interviews (important explanatory variables missing: education, family status, region of origin missing) | 21 | 21 | - | $P$ | Yes |
|  | **Eligible - Completed Interviews** | **1,572** | **1,094** | **478** | $\boldsymbol{I}$ | Yes |
|  | Estimated eligibility | 0.986 | 0.982 | 0.998 | $E$ |  |
|  | Response rate, lower bound | 0.639 | 0.560 | 0.958 | $RR_{4}$ |  |
|  | Response rate, upper bound | 0.926 | 0.897 | 1.000 | $RR_{6}$ |  |
|  | Response Rate, average | 0.782 | 0.728 | 0.979 | $RR_{4-6}$ |  |
|  | Adjusted response rate, upper bound | 0.681 | 0.600 | 1.000 | $RR_{6}^{'}$ |  |
|  | Adjusted Response Rate, average | 0.660 | 0.580 | 0.979 | $RR_{4-6}^{'}$ |  |
|  | Cooperation Rate | 0.913 | 0.880 | 1.000 | $COOP_{1}$ |  |
|  | Adjusted Cooperation rate | 0.672 | 0.589 | 1.000 | $COOP'_{1}$ |  |

# References

1. Refugee Studies Centre. Ethical guidelines for good research practice. Refugee Survey Quarterly. 2007;26(3):162-72. doi: 10.1093/rsq/hdi0250.

2. Clark-Kazak C. Developing ethical guidelines for research. Forced Migration Review. 2019;(61):12-4.

3. Buber-Ennser I, Kohlenberger J, Rengs B, Al Zalak Z, Goujon A, Striessnig E, et al. Human capital, values, and attitudes of persons seeking refuge in Austria in 2015. PLoS One. 2016;11(9):e0163481. doi: 10.1371/journal.pone.0163481.

4. Pędziwiatr K, Brzozowski J, Nahorniuk O. Refugees from Ukraine in Kraków (Poland). Cracow: Cracow University of Economics, 2022.

5. Behr D. Surveying the migrant population: Consideration of linguistic and cultural issues. Köln: GESIS; 2018.

6. Bloch A. Carrying out a survey of refugees: Some methodological considerations and guidelines. Journal of Refugee Studies. 1999;12(4):367-83. doi: 10.1093/jrs/12.4.367.

7. Müller-Funk L. Research with refugees in fragile political contexts: How ethical reflections impact methodological choices. Journal of Refugee Studies. 2021;34(2):2308-32. doi: 10.1093/jrs/feaa013.

8. State Statistics Service of Ukraine. Державна служба статистики України 2021. СТАТИСТИЧНИЙ ЗБІРНИК [Labour Force of Ukraine 2021. Statistical Yearbook]. Kyiv: State Statistics Service Ukraine; 2022.

9. Pędziwiatr K, Stonawski M, Brzozowski J. Immigrants in Cracow in 2021– Demographic Report. Kraków: Multicultural & Migration Observatory; 2021.

10. UMP. Miejska gościnność—Aktualizacja. Szacunek liczby Ukraincow w miastach UMP marzec, kwiecień, maj 2022 r [Urban Hospitality — Update. Estimation of the number of Ukrainians in the cities of the UMP March, April, May 2022]. Warsaw: Center of Analysis and Research of the Union of Polish Metropolises, 2022.

11. STATcube: Bevölkerung zu Jahresbeginn ab 2002 (einheitlicher Gebietsstand 2022) [STATcube: Population at the beginning of the year since 2002 (regional status of 2022)] [Internet]. Vienna: Statistics Austria. [cited 2022, 11 Nov]. Available from: <https://statcube.at/statcube/home>.

12. Bevölkerung nach Staatsangehörigkeit/Geburtsland [Population by nationality/country of birth] [Internet]. Vienna: Statistics Austria [cited 1 Sept 2022]. Available from: <https://www.statistik.at/statistiken/bevoelkerung-und-soziales/bevoelkerung/bevoelkerungsstand/bevoelkerung-nach-staatsangehoerigkeit/-geburtsland>.

13. Poland's Data Portal. Detailed statistics on persons registered in the register of citizens of Ukraine and members of their families [Internet]. [cited 2022, 18 Oct]. Available from: <https://dane.gov.pl/en/dataset/2715,zarejestrowane-wnioski-o-nadanie-statusu-ukr/resource/37804/table>.

14. American Association for Public Opinion Research. Standard definitions: Final dispositions of case codes and outcome rates for surveys. 7th edition. Washington: AAPOR; 2016.

15. Kohlenberger J, Rengs B, Buber-Ennser I. Nuclear family and social capital of refugees in Austria. International Migration. 2022;00. doi: 10.1111/imig.13073.
