# Supplementary material for: High self-selection of Ukrainian refugees into Europe: Evidence from Kraków and Vienna
Source: PLoS One. 2023 Dec 20;18(12):e0279783. doi: 10.1371/journal.pone.0279783 (PMC10732457; doi:10.1371/journal.pone.0279783)
Supplement: S2 File — English questionnaire. (PDF) [file pone.0279783.s002.pdf]

UkrPL

Ukrainian Arrivals in Poland

A Rapid-Response Survey on
Sociodemographic Characteristics, Needs and Resources

Questionnaire

This is a scientific survey whose purpose is to gain demographic information about Ukrainians who recently arrived in Poland. The project is conducted by independent academic researchers from the Cracow University of Economics. The participation in the survey is voluntary – you can resign from it at any time while filling the questionnaire!

Your answers will be treated strictly confidentially and anonymously. They are subject to data protection and statistical confidentiality. All results will be exclusively used for the purpose of the current survey.

Udział w badaniu nie ma wpływu na sytuację ankietowanego w Polsce, ani na jakość i zakres obsługi w Urzędzie Miasta Krakowa czy nabycie praw gwarantowanych ustawą z dnia 12 marca 2022 r. o pomocy obywatelom Ukrainy w związku z konfliktem zbrojnym na terytorium tego państwa.

This study is financed by the Cracow University of Economics and led by Dr. Elena Nahorniuk, Prof. UEK dr hab Konrad Pędziwiatr, Prof. UEK dr hab. Jan Brzozowski and Prof. UEK dr hab. Marcin Stonawski.

1 **What is your sex?**

- ☐ Female
- ☐ Male
- ☐ Other_________

2 **How old are you?**

__________ Years

3 **In which country were you born?**

- ☐ Ukraine
- ☐ Russia
- ☐ Other European country
- ☐ Non-European country

4 **What is your citizenship? Multiple answers are possible.**

- ☐ Ukraine
- ☐ Russia
- ☐ Other European country
- ☐ Non-European country

5 **Where in Ukraine have you lived most of your life?**

- ☐ Autonomous Republic of Crimea (before 2014)
- ☐ Cherkaska oblast
- ☐ Chernihivska oblast
- ☐ Chernivetska oblast
- ☐ Dnipropetrovska oblast
- ☐ Donetska oblast
- ☐ Ivano-Frankivska oblast
- ☐ Kharkivska oblast
- ☐ Khersonska oblast
- ☐ Khmelnytska oblast
- ☐ Kirovohradska oblast
- ☐ Kyiv
- ☐ Kyivska oblast
- ☐ Luhanska oblast
- ☐ Lvivska oblast
- ☐ Mykolaivska oblast
- ☐ Odeska oblast
- ☐ Poltavska oblast
- ☐ Rivnenska oblast
- ☐ Sumska oblast
- ☐ Ternopilska oblast
- ☐ Vinnytska oblast
- ☐ Volynska oblast
- ☐ Zakarpatska oblast
- ☐ Zaporizka oblast
- ☐ Zhytomyrska oblast

6 **What type of residence did you live in before you came here?**

- ☐ Your own house
- ☐ Your family's house
- ☐ Your own apartment
- ☐ A rented apartment
- ☐ A shared apartment with others
- ☐ Other

7 **How long did it take you to arrive to Poland from the place of your residence in Ukraine?**

__________ Days

8 **What is your religion?**

- ☐ Christian Orthodox
- ☐ Greek Catholic
- ☐ None (e.g. Atheist)
- ☐ Other religion

9 **What is your highest level of educational attainment?**

- ☐ Less than secondary general education
- ☐ Secondary general education (Atestat Pro Povnu Zagal´nu Serednyu Osvitu)
- ☐ Vocational education
- ☐ Bachelor (Bakalavr)
- ☐ Master (Magistr)
- ☐ Doctor of Philosophy, Arts or Science

10 **How many years of education have you completed?**

__________ Years

11 **Which language(s) do you speak**

- ☐ Ukrainian
- ☐ Russian
- ☐ English
- ☐ German
- ☐ Polish
- ☐ Hungarian
- ☐ Romanian
- ☐ French
- ☐ Spanish
- ☐ Portugese
- ☐ Other language(s)

11a **Which language(s) you mainly use in daily life** ____________________

12 **Did you ever actively participate in the labor market?**

- ☐ Yes → **go to question 13**
- ☐ No → **go to question 18**

13 **What is your main occupation?**

☞ Please enter ISCO 2-digit code from the list on the final page.

____

14 **Please enter the name/description of the occupation.**

____

15 **In which economic branch have you been active or employed?**

☞ Please enter NACE code from the list on the final page.

____

16 **Which best describes your work status before leaving Ukraine?**

- ☐ Employed
- ☐ Self-employed
- ☐ Working with/ for family member in a family business or a farm
- ☐ Student, in education or training
- ☐ Looking after home/family
- ☐ Unemployed
- ☐ Other

17 **How many hours did you usually work each week?**

- ☐ 1 to 9 hours
- ☐ 10 to 19 hours
- ☐ 20 to 34 hours
- ☐ 35 or more hours
- ☐ Seasonal work
- ☐ Hours vary considerably

18 **Do you plan to stay in Poland?**

- ☐ Yes → **go to question 20**
- ☐ Do not know → **go to question 20**
- ☐ No → **go to question 19**

19 **Where do you plan to go? Multiple answers are possible.**

- ☐ Germany
- ☐ Italy
- ☐ Switzerland
- ☐ Spain
- ☐ Sweden
- ☐ Portugal
- ☐ United Kingdom
- ☐ Austria
- ☐ Other European country
- ☐ Non-European country
- ☐ Back to Ukraine

20 **What do you want to do in Poland? Multiple answers are possible.**

- ☐ Search for a job → go to question 21
- ☐ To continue school/studying → go to question 22
- ☐ Wait until the war is over and live on savings and social benefits → go to question 22
- ☐ Do not know → go to question 22
- ☐ Do other things – what ____________________ → go to question 22

21 **How many hours do you want to work each week?**

☐ Less than 20 hours ☐ 20 to 30 hours ☐ 31 to 40 hours ☐ 41 or more hours

22 **What is your general assessment of your health?**

☐ Very good ☐ Good ☐ Neither good nor bad
☐ Bad ☐ Very bad

23 **Do you have any long-term (chronic) illness or health problems?**

☐ Yes ☐ No

24 **For the past six months at least, to what extent have you been limited because of a health problem in activities people usually do?**

☐ Very much ☐ To some extent ☐ Not at all

25 **In times of trouble, can you count on at least some of your family or friends?**

☐ Yes, very much ☐ Yes, to some extent ☐ No, not at all

26 **Are you regularly seeking support in (online) spiritual events or praying to God regularly?**

☐ Yes, very much ☐ Yes, to some extent ☐ No, not at all

27 **Are you regularly seeking support in (online) community organization (Facebook, Telegram groups for Ukrainian refugees)?**

☐ Yes, very much ☐ Yes, to some extent ☐ No, not at all

28 **Did you feel that you were welcome when you arrived in Poland?**

☐ Totally ☐ Mostly ☐ In some ways
☐ Hardly at all ☐ Not at all ☐ Do not know

29 **When did you arrive in Poland?**

______________________________ (Day/Month/Year)

30 **How did you come to Poland?**

☐ Directly ☐ Trough Slovakia
☐ Through Hungary ☐ Through Romania
☐ Through Moldova ☐ Through another country

31 **Why did you decide to flee from the war to Poland? Multiple answers are possible.**

- ☐ By chance/ not planned/ just ended up here
- ☐ I have family here
- ☐ I have friends here
- ☐ I know people here related to my/my partner’s work
- ☐ Because it is easier to find work here
- ☐ Because it is close to Ukraine and I want to go back immediately when the war ends

Other ____

32 **Which statements below may express your considerations about returning to Ukraine?**

- ☐ I have nothing to return to in Ukraine
- ☐ I want to return as soon as the war ends
- ☐ I may return in case the war ends
- ☐ I may return even if the war continues
- ☐ I do not have an idea, I do not know

33 **What is your family status?**

- ☐ Married → **go to question 34**
- ☐ Cohabitation with partner → **go to question 34**
- ☐ Widowed → **go to question 36**
- ☐ Divorced → **go to question 36**
- ☐ Single → **go to question 36**

34 **How old is your partner?**

__________ Years

35 **Where does your partner currently live?**

- ☐ He/she is with me
- ☐ Ukraine
- ☐ Other country
- ☐ Do not know

36 **How many children do you have?**

- ☐ No children → **go to question 43**

__________ Children → **go to question 37**

37-42 **Can you specify some details about your children?**

	**37** **Child 1**	**38** **Child 2**	**39** **Child 3**
**Age**	☐ Less than 1 year<br>_________ Years	☐ Less than 1 year<br>_________ Years	☐ Less than 1 year<br>_________ Years
**Sex**	☐ Male<br>☐ Female	☐ Male<br>☐ Female	☐ Male<br>☐ Female
**Where does he/she currently live?**	☐ Here with me<br>☐ Ukraine<br>☐ Other country<br>☐ Do not know	☐ Here with me<br>☐ Ukraine<br>☐ Other country<br>☐ Do not know	☐ Here with me<br>☐ Ukraine<br>☐ Other country<br>☐ Do not know
**How many years did he/she go to school?**	__________ Years	__________ Years	__________ Years

	40 Child 4	41 Child 5	42 Child 6
**Age**	☐ Less than 1 year<br>_________ Years	☐ Less than 1 year<br>_________ Years	☐ Less than 1 year<br>_________ Years
**Sex**	☐ Male<br>☐ Female	☐ Male<br>☐ Female	☐ Male<br>☐ Female
**Where does he/she currently live?**	☐ Here with me<br>☐ Ukraine<br>☐ Other country<br>☐ Do not know	☐ Here with me<br>☐ Ukraine<br>☐ Other country<br>☐ Do not know	☐ Here with me<br>☐ Ukraine<br>☐ Other country<br>☐ Do not know
**How many years did he/she go to school?**	__________ Years	__________ Years	__________ Years

43 Do you currently expect a child?

☐ Yes ☐ No ☐ Prefer not to answer

44 Did you arrive in Poland with other family members, friends or neighbors? Multiple answers are possible.

☐ Mother
☐ Mother in law
☐ Cousin(s)
☐ Friend(s)
☐ With my children
☐ Father
☐ Father in law
☐ Nephew(s)/ niece(s)
☐ Other acquaintances
☐ Sister(s)
☐ Brother(s)
☐ Neighbor(s)
☐ Other person/s – who? _______________

45 In what type of accommodation do you currently live here in Poland?

☐ Rented single room
☐ Rented apartment
☐ Rented house
☐ Own apartment
☐ Own house
☐ Living in the house or apartment with Polish family
☐ Living in the house or apartment of someone for which I don't have to pay
☐ Temporary shelter exchanged among refugees
☐ Collective Shelter
☐ Other

46 How do you feel in your neighbourhood in Poland?

☐ Safe ☐ Somewhat safe ☐ Somewhat unsafe ☐ Unsafe

47 If you do not feel safe, why? Multiple answers are possible.

☐ Racism/discrimination
☐ Theft
☐ Place where I stay is unsafe
☐ Threats of violence/verbal assault
☐ Fear of closing the shelter or camp
☐ Other
☐ Problems with Polish locals
☐ Some bad individuals
☐ Poland is not safe
☐ Lack of proper shelter
☐ Fear of other ethnic/religious groups

48 **What do you think about a democratic political system as a way of governing Ukraine? Would you say it is a very good, fairly good, fairly bad or very bad way of governing?**

☐ Very good
☐ Fairly good
☐ Fairly bad
☐ Very bad

49 **How much confidence do you have in the European Union? Is it a great deal of confidence, quite a lot of confidence, not very much confidence or none at all?**

☐ A great deal
☐ Quite a lot
☐ Not very much
☐ None at all

50 **Do you feel safe in Poland as a NATO country?**

☐ Yes, very much so
☐ Rather yes
☐ Neither yes nor no
☐ Rather no
☐ Surely no

51 **Should NATO intervene in Ukraine?**

☐ Yes, immediately
☐ Yes but only when chemical weapons are used
☐ Yes but only when biological weapons are used
☐ Yes but only when tactical nuclear weapons are used
☐ NATO should stay away from direct involvement in the war

52 **What is the most important support you were offered in Poland? (multiple answers possible)?**

☞ Serious problem (1), A minor problem (2), No problem (3), Not relevant (0)

**Support offered**	**Who offered support** [choose from the list A below]
A shelter (a place to stay)	____
Means of subsistence (food/water, clothing etc)	____
Logistic support to reach your destination such as the camp, relatives, or elsewhere	____
Legal assistance about your status 5. Information	____
Information	____
Other (please briefly specify) ____________________	____
No support was offered	____
No support was needed	____

List A:

1. Local individuals
2. Relatives or friends of yours
3. Police/soldiers/border guards
4. Public institutions
5. Local humanitarian organizations
6. International humanitarian
7. Religious institutions (churches)
8. Other

53 **Below you can find a list of issues related to the protection situation of refugees. For each one, please mark if it is a serious problem, a minor problem, or no problem at all for you and your family in Poland?**

☞ Serious problem (1), A minor problem (2), No problem (3), Not relevant (0)

Access to labour market/ availability of jobs	1	2	3	0
Access to medical care	1	2	3	0
Access to psychological care	1	2	3	0
Access to financial support	1	2	3	0
Access to education	1	2	3	0
Access to adequate housing	1	2	3	0
Access to residence permit	1	2	3	0
Access to legal aid	1	2	3	0
Access to security forces or court case when you encounter problems	1	2	3	0
Protection against exploitation in work place	1	2	3	0
Lack of safety	1	2	3	0
Preconceptions and misconceptions about refugees	1	2	3	0

54 **Are you learning Polish**

☐ Yes → **go to question 57**

☐ No → **go to question 55**

55 **If you don't, why?**

☐ I don't find it necessary

☐ Too difficult

☐ Other reasons (please specify) ____

☐ I speak Polish

56 **Do you wish to learn Polish?**

☐ Yes

☐ No

57 **Have you ever had a paid job in Poland, either as an employee or as self-employed?**

☐ Yes

☐ No

58 **Are you currently working?**

☐ Yes → **go to question 59**

☐ No → **go to question 66**

59 **How long did it take you to start working after your arrival in Poland?**

______________________________ (Days)

60 **What is your current job performed in Poland?**

- ☐ Unskilled worker (e.g. maid, waiter, kitchen help, agricultural worker, cleaner, babysitter)
- ☐ Skilled worker or craftsman (e.g. welder, machine operator, qualified bricklayer, tailor, nurse, operator of agricultural machinery, forester)
- ☐ Service employee or salesperson (hairdresser, beautician, cook)
- ☐ Office worker, a technician and other middle personnel (secretary, electrician)
- ☐ Specialist (lawyer, doctor, bookkeeper, lecturer, IT specialist, teacher, translator)
- ☐ Manager/supervisor/director
- ☐ Other (please specify) ___________________

61 **In which sector is your current job performed in Poland?.**

- ☐ Agriculture
- ☐ Manufacturing (industry and crafts)
- ☐ Retail/ wholesale trade
- ☐ Tourism
- ☐ Foodservice
- ☐ Construction and renovation services
- ☐ Household services
- ☐ Education and translation
- ☐ Health and social service
- ☐ IT/banking/accounting/consulting/marketing
- ☐ Other (please specify) ___________________

62 **Is your current job performed in Poland:**

- ☐ Below your qualifications
- ☐ Above your qualifications
- ☐ At the same level qualifications as a job performed in Ukraine

63 **How did you find your current job?**

- ☐ Through family or friends
- ☐ Through NGO
- ☐ Through official institution (e.g. Job center)
- ☐ Through intermediaries
- ☐ Another way (how?) ______________________________________

64 **How many hours per week do you work currently?**

____________________________ (Hours weekly)

65 **What level of Polish is required for your job?**

- ☐ No proficiency
- ☐ Only basic communication skills
- ☐ Good command/ good working knowledge
- ☐ Very good command
- ☐ Excellent command/ highly proficient in spoken and written
- ☐ Near native/ fluent

66 **To what extent here in Poland do the following help you in coping with any difficult situation you are facing?**

☞ Where “99” means “not relevant”, “1” means not at all and “10” means very much Not at all Very much Not relevant.

Family	1 2 3 4 5 6 7 8 9 10 99
Friends	1 2 3 4 5 6 7 8 9 10 99
Faith/ Religion/ Spirituality	1 2 3 4 5 6 7 8 9 10 99
Work /school	1 2 3 4 5 6 7 8 9 10 99
Social/institutional benefits system	1 2 3 4 5 6 7 8 9 10 99
Being out in Nature (e.g. in the wood, at the park)	1 2 3 4 5 6 7 8 9 10 99
	1 2 3 4 5 6 7 8 9 10 99

Please add to the list any important aspects that you find in Poland.

Other (please briefly specify)

____________________________________ 1 2 3 4 5 6 7 8 9 10 99

NACE-Codes

A Agriculture, forestry and fishing
B Mining and quarrying
C Manufacturing
D Electricity, gas, steam and air conditioning supply
E Water supply; sewerage; waste management and remediation activities
F Construction
G Wholesale and retail trade; repair of motor vehicles and motorcycles
H Transporting and storage
I Accommodation and food service activities
J Information and communication
K Financial and insurance activities
L Real estate activities
M Professional, scientific and technical activities
N Administrative and support service activities
O Public administration and defense; compulsory social security
P Education
Q Human health and social work activities
R Arts, entertainment and recreation
S Other services activities
T Activities of households as employers; undifferentiated goods - and services - producing activities of households for own use
U Activities of extraterritorial organizations and bodies

ISCO-Codes

0 Armed forces occupations

1 Managers
11 Chief executives, senior officials and legislators
12 Administrative and commercial managers
13 Production and specialized services managers
14 Hospitality, retail and other services managers

2 Professionals
21 Science and engineering professionals
22 Health professionals
23 Teaching professionals
24 Business and administration professionals
25 Information and communications technology professionals
26 Legal, social and cultural professionals

3 Technicians and associate professionals
31 Science and engineering associate professionals
32 Health associate professionals
33 Business and administration associate professionals
34 Legal, social, cultural and related associate professionals
35 Information and communications technicians

4 Clerical support workers
41 General and keyboard clerks
42 Customer services clerks
43 Numerical and material recording clerks
44 Other clerical support workers

5 Service and sales workers
51 Personal service workers
52 Sales workers
53 Personal care workers
54 Protective services workers

6 Skilled agricultural, forestry and fishery workers
61 Market-oriented skilled agricultural workers
62 Market-oriented skilled forestry, fishery and hunting workers
63 Subsistence farmers, fishers, hunters and gatherers

7 Craft and related trades workers
71 Building and related trades workers, excluding electricians
72 Metal, machinery and related trades workers
73 Handicraft and printing workers
74 Electrical and electronics trades workers
75 Food processing, wood working, garment and other craft and related trades workers

8 Plant and machine operators, and assemblers
81 Stationary plant and machine operators
82 Assemblers
83 Drivers and mobile plant operators

9 Elementary occupations
91 Cleaners and helpers
92 Agricultural, forestry and fishery laborers
93 Laborers in mining, construction, manufacturing and transport
94 Food preparation assistants
95 Street and related sales and service workers
96 Refuse workers and other elementary workers
