# Supplementary material for: High self-selection of Ukrainian refugees into Europe: Evidence from Kraków and Vienna
Source: PLoS One. 2023 Dec 20;18(12):e0279783. doi: 10.1371/journal.pone.0279783 (PMC10732457; doi:10.1371/journal.pone.0279783)
Supplement: S3 File — English questionnaire. (PDF) [file pone.0279783.s003.pdf]

UkrAiA

Ukrainian Arrivals in Austria

A Rapid-Response Survey on Sociodemographic Characteristics, Needs and Resources

Vienna University of Economics and Business
Welthandelsplatz 1, 1020 Vienna

Vienna Institute of Demography/Austrian Academy of Sciences
Vordere Zollamtsstraße 3, 1030 Vienna

Salzburg University of Applied Sciences
Urstein Süd 1, 5412 Puch

Questionnaire

This is a scientific survey whose purpose is to gain demographic information about Ukrainians who recently arrived in Austria. The project is conducted by independent academic researchers from the Vienna University of Economics and Business and the Austrian Academy of Sciences.

Your answers will be treated strictly confidentially and anonymously. They are subject to data protection and statistical confidentiality. All results will be exclusively used for the purpose of the current survey.

This study is co-financed by the City of Vienna (MA17) and the Vienna Social Fund (FSW).

1 **What is your sex?**

☐ Female ☐ Male ☐ Divers

2 **How old are you?**

__________ Years

3 **In which country were you born?**

☐ Ukraine ☐ Russia ☐ Other European country ☐ Non-European country

4 **Which country was your last place of residence?**

☐ Ukraine ☐ Other country

5 **What is your citizenship? Multiple answers are possible.**

☐ Ukraine ☐ Russia ☐ Other European country ☐ Non-European country

6 **Where in Ukraine have you lived most of your life?**

☐ Autonomous Republic of Crimea (before 2014)
☐ Cherkaska oblast
☐ Chernihivska oblast
☐ Chernivetska oblast
☐ Dnipropetrovska oblast
☐ Donetska oblast
☐ Ivano-Frankivska oblast
☐ Kharkivska oblast
☐ Khersonska oblast
☐ Khmelnytska oblast
☐ Kirovohradska oblast
☐ Kyiv
☐ Kyivska oblast
☐ Luhanska oblast
☐ Lvivska oblast
☐ Mykolaivska oblast
☐ Odeska oblast
☐ Poltavska oblast
☐ Rivnenska oblast
☐ Sumska oblast
☐ Ternopilska oblast
☐ Vinnytska oblast
☐ Volynska oblast
☐ Zakarpatska oblast
☐ Zaporizka oblast
☐ Zhytomyrska oblast

7 **What type of residence did you live in before you came here?**

☐ Your own house
☐ Your family's house
☐ Your own apartment
☐ A rented apartment
☐ A shared apartment with others
☐ Other

8 **What is your religion?**

☐ Christian Orthodox
☐ Greek Catholic
☐ None (e.g. Atheist)
☐ Other religion

9 **What is your highest level of educational attainment?**

☐ Less than secondary general education
☐ Secondary general education (Atestat Pro Povnu Zagal´nu Serednyu Osvitu)
☐ Vocational education
☐ Bachelor (Bakalavr)
☐ Master (Magistr)
☐ Doctor of Philosophy, Arts or Science

10 **How many years of education have you completed?**

__________ Years

11 **Which language(s) do you speak?**

☐ Ukrainian	☐ Russian	☐ English	☐ German
☐ Polish	☐ Hungarian	☐ Romanian	☐ French
☐ Spanish	☐ Portugese	☐ Other language(s)	

12 **Did you ever actively participate in the labor market?**

☐ Yes → **go to question 13**
☐ No → **go to question 18**

13 **What is your main occupation?**

☞ Please enter ISCO 2-digit code from the list on the final page.

____

14 **Please enter the name/description of the occupation.**

____

15 **In which economic branch have you been active or employed?**

☞ Please enter NACE code from the list on the final page.

____

16 **Which best describes your work status before leaving the country?**

☐ Employed
☐ Self-employed
☐ Working with/ for family member in a family business or a farm
☐ Student, in education or training
☐ Looking after home/family
☐ Unemployed
☐ Other

17 **How many hours did you usually work each week?**

☐ 1 to 9 hours	☐ 35 or more hours
☐ 10 to 19 hours	☐ Seasonal work
☐ 20 to 34 hours	☐ Hours vary considerably

18 **Have you completed any kind of training in nursing or care?**

☐ Yes, with academic graduation	☐ Yes, without graduation
☐ Yes, with non-academic graduation	☐ No

19 **Have you ever worked in nursing or care?**

☐ Yes

__________ Years in general care

__________ Years in geriatric care

__________ Years in pedriatic care

☐ No

20 **Are you interested to start or continue a training in nursing/care in Austria?**

☐ Yes ☐ No

21 **Do you plan to stay in Austria?**

- ☐ Yes → go to question 23
- ☐ Do not know → go to question 23
- ☐ No → go to question 22

22 **Where do you plan to go? Multiple answers are possible.**

- ☐ Germany
- ☐ Italy
- ☐ Switzerland
- ☐ Spain
- ☐ Sweden
- ☐ Portugal
- ☐ United Kingdom
- ☐ Czech Republic
- ☐ Other European country
- ☐ Non-European country

23 **What do you want to do in Austria?**

- ☐ Search for a job → go to question 24
- ☐ To continue school/studying → go to question 25
- ☐ Do not know → go to question 25

24 **How many hours do you want to work each week?**

- ☐ Less than 20 hours
- ☐ 20 to 34 hours
- ☐ 35 or more hours

25 **What is your general assessment of your health?**

- ☐ Very good
- ☐ Good
- ☐ Neither good nor bad
- ☐ Bad
- ☐ Very bad

26 **Do you have any long-term (chronic) illness or health problems?**

- ☐ Yes
- ☐ No

27 **For the past six months at least, to what extent have you been limited because of a health problem in activities people usually do?**

- ☐ Very much
- ☐ To some extent
- ☐ Not at all

28 **In times of trouble, can you count on at least some of your family or friends?**

- ☐ Yes, very much
- ☐ Yes, to some extent
- ☐ No, not at all

29 **Are you regularly seeking support in (online) spiritual events or praying to god regularly?**

- ☐ Yes, very much
- ☐ Yes, to some extent
- ☐ No, not at all

30 **Are you regularly seeking support in (online) community organization (Facebook, Telegram groups for Ukrainian refugees)?**

- ☐ Yes, very much
- ☐ Yes, to some extent
- ☐ No, not at all

31 **Did you feel that you were welcome when you arrived in Austria?**

- ☐ Totally
- ☐ Mostly
- ☐ In some ways
- ☐ Hardly at all
- ☐ Not at all
- ☐ Do not know

32 **When did you arrive in Austria?**

______________________________ (Day/Month/Year)

33 **How long did it take you to arrive in Austria?**

__________ Days

34 How did you come to Austria? Multiple answers are possible.

☐ Through Poland	☐ Trough Slovakia
☐ Through Hungary	☐ Through Romania
☐ Through Czechia	☐ Through another country

35 Why did you flee to Austria? Multiple answers are possible.

- ☐ By chance/ not planned/ just ended up here
- ☐ I have family here
- ☐ I have friends here
- ☐ I know people here related to my/my partner’s work
- ☐ Because it is easier to find work here
- ☐ Because the social welfare benefits are better here
- ☐ Because the medical care is better here

Other ____

36 How much did your journey to Austria cost (per person, in Ukrainian hryvnia)?

☐ < 3.000 ₴	☐ >=3.000 ₴ < 6.000 ₴	☐ > 6.000 ₴
☐ No costs	☐ Do not know	

37 Which statements below may express your considerations about returning to Ukraine?

- ☐ I have nothing to return to in Ukraine
- ☐ I want to return as soon as the war ends
- ☐ I may return in case the war ends
- ☐ I may return even if the war continues
- ☐ I do not have an idea, I do not know

38 What is your family status?

☐ Married	→ **go to question 39**
☐ Cohabitation with partner	→ **go to question 39**
☐ Widowed	→ **go to question 47**
☐ Divorced	→ **go to question 47**
☐ Single	→ **go to question 47**

39 How old is your partner?

__________ Years

40 Where does your partner currently live?

☐ He/she is with me	☐ Other country
☐ Ukraine	☐ Do not know

41 What is your partner’s highest level of educational attainment?

- ☐ Less than secondary general education
- ☐ Secondary general education (Atestat Pro Povnu Zagal´nu Serednyu Osvitu)
- ☐ Vocational education
- ☐ Bachelor (Bakalavr)
- ☐ Master (Magistr)
- ☐ Doctor of Philosophy, Arts or Science
- ☐ Do not know

42 **Did your partner ever actively participate in the labor market?**

- ☐ Yes
- ☐ No → go to question 47
- ☐ Do not know → go to question 47

43 **What is your partner's main occupation?**

☞ Please enter ISCO 2-digit code from the list on the final page.

____

44 **Please enter the name/description of your partner's occupation.**

____

45 **In which economic branch is/was your partner active or employed?**

☞ Please enter NACE code from the list on the final page.

____

46 **Which best describes the work status of your partner before the beginning of the war?**

- ☐ Employed
- ☐ Self-employed
- ☐ Working with/ for family member in a family business or a farm
- ☐ Student
- ☐ Looking after home/family
- ☐ Unemployed
- ☐ Other
- ☐ Do not know

47 **How many children do you have or have had (including deceased children)?**

- ☐ No children → go to question 54

__________ Children → go to question 48

48-53 **Can you specify some details about your children?**

	48 **Child 1**	49 **Child 2**	50 **Child 3**
**Age**	☐ Less than 1 year<br>_________ Years	☐ Less than 1 year<br>_________ Years	☐ Less than 1 year<br>_________ Years
**Sex**	☐ Male<br>☐ Female	☐ Male<br>☐ Female	☐ Male<br>☐ Female
**Where does he/she currently live?**	☐ Here with me<br>☐ Ukraine<br>☐ Other country<br>☐ Deceased<br>☐ Do not know	☐ Here with me<br>☐ Ukraine<br>☐ Other country<br>☐ Deceased<br>☐ Do not know	☐ Here with me<br>☐ Ukraine<br>☐ Other country<br>☐ Deceased<br>☐ Do not know
**How many years did he/she go to school?**	__________ Years	__________ Years	__________ Years

	51 Child 4	52 Child 5	53 Child 6
**Age**	☐ Less than 1 year<br>_________ Years	☐ Less than 1 year<br>_________ Years	☐ Less than 1 year<br>_________ Years
**Sex**	☐ Male<br>☐ Female	☐ Male<br>☐ Female	☐ Male<br>☐ Female
**Where does he/she currently live?**	☐ Here with me<br>☐ Ukraine<br>☐ Other country<br>☐ Deceased<br>☐ Do not know	☐ Here with me<br>☐ Ukraine<br>☐ Other country<br>☐ Deceased<br>☐ Do not know	☐ Here with me<br>☐ Ukraine<br>☐ Other country<br>☐ Deceased<br>☐ Do not know
**How many years did he/she go to school?**	__________ Years	__________ Years	__________ Years

54 **Do you currently expect a child?**

☐ Yes ☐ No

55 **Did you arrive in Austria with other family members, friends or neighbors? Multiple answers are possible.**

☐ Mother
☐ Father
☐ Sister(s)
☐ Mother in law
☐ Father in law
☐ Brother(s)
☐ Cousin(s)
☐ Nephew(s)/ niece(s)
☐ Neighbor(s)
☐ Friend(s)
☐ Other acquaintances

56 **In what type of accommodation do you currently live here in Austria?**

☐ Rented single room
☐ Rented apartment
☐ Rented house
☐ Own apartment
☐ Own house
☐ Living in the house or apartment with Austrian family
☐ Temporary shelter exchanged among refugees
☐ Collective Shelter
☐ Other

57 **How do you feel in your neighbourhood in Austria?**

☐ Safe ☐ Somewhat safe ☐ Somewhat unsafe ☐ Unsafe

58 **If you do not feel safe, why? Multiple answers are possible.**

☐ Racism/discrimination
☐ Problems with Austrian locals
☐ Theft
☐ Some bad individuals
☐ Place where I stay is unsafe
☐ Austria is not safe
☐ Threats of violence/verbal assault
☐ Lack of proper shelter
☐ Fear of closing the shelter or camp
☐ Fear of other ethnic/religious groups
☐ Other

The following statements were selected from the World Value Survey, a scientific investigation which seeks to find out about the beliefs, values and motivations of people throughout the world. There is no “right” or “wrong” answer to these questions.

Please note that we do not ask for your country’s official or legal stance on the following statements nor should your response be guided by social conventions and expectations. Rather, we are interested in your personal beliefs and values.

We would like to stress that your answers to these questions are completely confidential, our survey is conducted for purely scientific purposes and none of the data you provide us with will be forwarded to third parties.

59 **When jobs are scarce, men should have more rights to a job than women.**

- ☐ Strongly agree
- ☐ Agree
- ☐ Neither agree/nor disagree
- ☐ Disagree
- ☐ Strongly disagree

60 **On the whole, men make better business executives than women do.**

- ☐ Strongly agree
- ☐ Agree
- ☐ Disagree
- ☐ Strongly disagree

61 **Being a housewife is just as fulfilling as working for pay.**

- ☐ Strongly agree
- ☐ Agree
- ☐ Disagree
- ☐ Strongly disagree

62 **What do you think about having a democratic political system as a way of governing Ukraine? Would you say it is a very good, fairly good, fairly bad or very bad way of governing?**

- ☐ Very good
- ☐ Fairly good
- ☐ Fairly bad
- ☐ Very bad

63 **How much confidence do you have in the European Union? Is it a great deal of confidence, quite a lot of confidence, not very much confidence or none at all?**

- ☐ A great deal
- ☐ Quite a lot
- ☐ Not very much
- ☐ None at all

64 **How much confidence do you have in the North Atlantic Treaty Organization (NATO)? A great deal of confidence, quite a lot of confidence, not very much confidence or none at all?**

- ☐ A great deal
- ☐ Quite a lot
- ☐ Not very much
- ☐ None at all

65 **Apart from the fact of belonging to a religious community or not, how religious do you consider yourself?**

☞ Please mark the appropriate number, where 0 indicates "not at all religious" and 10 "very religious".

Not at all religious										Very religious
0	1	2	3	4	5	6	7	8	9	10

66 **People sometimes describe themselves as belonging to the working class, the middle class, or the upper or lower class. Would you describe yourself as belonging to the**

- ☐ Upper class
- ☐ Upper middle class
- ☐ Lower middle class
- ☐ Working class
- ☐ Lower class

67 **Below is an income scale on which 1 indicates the lowest income group and 10 the highest income group in your country. We would like to know in what group your household is.**

☞ Please mark the appropriate number, counting all wages, salaries, pensions and other incomes that come in.

Lowest group									Highest group
1	2	3	4	5	6	7	8	9	10

68 **We have now come to the end of our interview. Thank you very much for your participation! We wish you all the best for your future. May we interview you again in the future?**

☐ Yes ☐ No

69 **Would you like to be contacted in case of job offers, based on the qualifications and professional experience you provided?**

☐ Yes ☐ No

70 **Could you please give us your email address, Facebook address, other social network account name, telephone number (for Telegram, Whatsapp or Signal) or your name so that we can contact you?**

Your contact details ____

We will ensure that your answers are only accessed by authorized and verified researchers for scientific purposes.

If you have any questions about the survey or would like further information, you can contact us at +43-(0)1-31336-4847 or by emailing survey@ukraia.at.

NACE-Codes

A Agriculture, forestry and fishing
B Mining and quarrying
C Manufacturing
D Electricity, gas, steam and air conditioning supply
E Water supply; sewerage; waste management and remediation activities
F Construction
G Wholesale and retail trade; repair of motor vehicles and motorcycles
H Transporting and storage
I Accommodation and food service activities
J Information and communication
K Financial and insurance activities
L Real estate activities
M Professional, scientific and technical activities
N Administrative and support service activities
O Public administration and defense; compulsory social security
P Education
Q Human health and social work activities
R Arts, entertainment and recreation
S Other services activities
T Activities of households as employers; undifferentiated goods - and services - producing activities of households for own use
U Activities of extraterritorial organizations and bodies

ISCO-Codes

0 Armed forces occupations

1 Managers
11 Chief executives, senior officials and legislators
12 Administrative and commercial managers
13 Production and specialized services managers
14 Hospitality, retail and other services managers

2 Professionals
21 Science and engineering professionals
22 Health professionals
23 Teaching professionals
24 Business and administration professionals
25 Information and communications technology professionals
26 Legal, social and cultural professionals

3 Technicians and associate professionals
31 Science and engineering associate professionals
32 Health associate professionals
33 Business and administration associate professionals
34 Legal, social, cultural and related associate professionals
35 Information and communications technicians

4 Clerical support workers
41 General and keyboard clerks
42 Customer services clerks
43 Numerical and material recording clerks
44 Other clerical support workers

5 Service and sales workers
51 Personal service workers
52 Sales workers
53 Personal care workers
54 Protective services workers

6 Skilled agricultural, forestry and fishery workers
61 Market-oriented skilled agricultural workers
62 Market-oriented skilled forestry, fishery and hunting workers
63 Subsistence farmers, fishers, hunters and gatherers

7 Craft and related trades workers
71 Building and related trades workers, excluding electricians
72 Metal, machinery and related trades workers
73 Handicraft and printing workers
74 Electrical and electronics trades workers
75 Food processing, wood working, garment and other craft and related trades workers

8 Plant and machine operators, and assemblers
81 Stationary plant and machine operators
82 Assemblers
83 Drivers and mobile plant operators

9 Elementary occupations
91 Cleaners and helpers
92 Agricultural, forestry and fishery laborers
93 Laborers in mining, construction, manufacturing and transport
94 Food preparation assistants
95 Street and related sales and service workers
96 Refuse workers and other elementary workers
