# Supplementary material for: High self-selection of Ukrainian refugees into Europe: Evidence from Kraków and Vienna
Source: PLoS One. 2023 Dec 20;18(12):e0279783. doi: 10.1371/journal.pone.0279783 (PMC10732457; doi:10.1371/journal.pone.0279783)
Supplement: S1 Table — Sources: UkrPL and UkrAiA. (PDF) [file pone.0279783.s004.pdf]

S1 Table. Sample characteristics, in %.

	Kraków	Vienna
Gender		
Male	3	11
Female	97	89
Age		
18-24	9	13
25-34	26	25
35-44	40	33
45+	25	28
Mean age in years	39 years	39 years
Country of birth		
Ukraine	94	93
Russia	2	4
Other country	4	3
Part of Ukraine living most of the life		
Kyiv	16	30
Central Ukraine	18	13
Western Ukraine	16	17
Southern Ukraine	26	23
Eastern Ukraine	23	17
Type of residence before leaving Ukraine		
Own house	33	26
Own apartment	58	58
Rented apartment	7	12
Shared apartment	0	3
Other	2	2
Highest level of education		
Secondary general education or less	19	9
Vocational education	16	10
Bachelor degree	23	26
Master degree	39	51
PhD	3	3
Language skills		
Ukrainian	99	99
Russian	86	90
English	34	62
German	2	15
Polish	21	8
Relationship status		
Married	51	51
Cohabitation with partner	8	10
Widowed	5	4
Divorced	16	11
Single	18	23
No answer	1	0
Current place of partner*		
He/she is with me	20	37
Ukraine	72	58
Other country	6	4
Do not know	1	0
No answer	1	1
Parity		
Childless	30	39
1 child	36	31
2 children	27	23
3 children	5	6
4+ children	2	1
Mean number of children	1.1 children	1.0 children
Mean number of children among parents	1.6 children	1.6 children
Current pregnancy		
Yes	2	2
No	81	86
No answer	17	12
Time of arrival		
February 2022	13	13
1st half of March 2022	57	43
2nd half of March 2022	10	21
April-June 2022	17	23
No answer	2	0
Average number of days to arrive in Poland/Austria	3 days	4 days
Interview method		
PAPI	100	92
CAWI	0	8
N	472	1,094

Sources: UkrPL and UkrAiA. Note: *Sub-sample of respondents in a partnership (Kraków: N=280; Vienna: N=671).
