# Supplementary material for: High self-selection of Ukrainian refugees into Europe: Evidence from Kraków and Vienna
Source: PLoS One. 2023 Dec 20;18(12):e0279783. doi: 10.1371/journal.pone.0279783 (PMC10732457; doi:10.1371/journal.pone.0279783)
Supplement: S2 Table — Sources: UkrPL and UkrAiA. (PDF) [file pone.0279783.s005.pdf]

S2 Table. Previous employment and planned labour market participation in host country, persons aged 18-59 years, in %.

	Women			Men		
	Kraków	Vienna	Vienna incl. partners	Kraków	Vienna	Vienna incl. partners
**Ever participated in labour market**						
Yes	84.1	90.4	89.8	(90.9)	82.3	86.3
No	15.5	7.7	8.2	(9.1)	16.7	10.3
Don’t know/no answer	0.5	1.9	2.0	(0.0)	1.0	3.4
**Work status before leaving the country**						
Employed	48.7	51.9	51.4	(45.5)	37.5	45.5
Self-employed	12.5	21.2	20.6	(27.3)	30.2	25.3
Working in a family business or a farm	2.1	1.7	1.7	(9.1)	1.0	3.4
Student, in education or training	1.8	2.0	1.9	(0.0)	3.1	1.7
Looking after home/family	10.6	6.5	6.7	(0.0)	3.1	1.7
Unemployed	4.6	3.8	4.0	(0.0)	2.1	3.4
Other/no answer	3.7	3.3	3.5	(0.0)	5.2	5.2
Never participated in LM	15.9	9.6	10.2	(9.1)	17.7	13.7
N	433	899	965	(11)	96	233

Sources: UkrPL and UkrAiA.

Note: Columns “incl. partners” include partners living in Austria/Vienna at the time of the survey. Percentages in parentheses indicate low case numbers.
