# Supplementary material for: High self-selection of Ukrainian refugees into Europe: Evidence from Kraków and Vienna
Source: PLoS One. 2023 Dec 20;18(12):e0279783. doi: 10.1371/journal.pone.0279783 (PMC10732457; doi:10.1371/journal.pone.0279783)
Supplement: S3 Table — Sources: State Statistics Service of Ukraine [73]. (PDF) [file pone.0279783.s006.pdf]

S3 Table. Employment in Ukraine, 2021, in %.

	Persons aged 15-70			Persons aged 15-59		
	Women	Men	Total	Women	Men	Total
Employed	50.4	61.5	55.7	60.7	69.9	65.3
Unemployed	5.7	6.5	6.1	7.2	7.7	7.5
Out of labour force	43.9	31.9	38.2	32.1	22.3	27.3
Total	100	100	100	100	100	100

Sources: State Statistics Service of Ukraine [51].
