# Supplementary material for: High self-selection of Ukrainian refugees into Europe: Evidence from Kraków and Vienna
Source: PLoS One. 2023 Dec 20;18(12):e0279783. doi: 10.1371/journal.pone.0279783 (PMC10732457; doi:10.1371/journal.pone.0279783)
Supplement: S4 Table — Sources: State Statistics Service of Ukraine [73]. (PDF) [file pone.0279783.s007.pdf]

S4 Table. Employment status of persons aged 15-70 years, Ukraine, 2021, in %.

Employed	46.6
Self-employed	9.0
Contributing family workers	0.1
Unemployed	6.1
Out of labour force	38.2
Total	100

Sources: State Statistics Service of Ukraine [51].
