# Supplementary material for: High self-selection of Ukrainian refugees into Europe: Evidence from Kraków and Vienna
Source: PLoS One. 2023 Dec 20;18(12):e0279783. doi: 10.1371/journal.pone.0279783 (PMC10732457; doi:10.1371/journal.pone.0279783)
Supplement: S5 Table — Sources: State Statistics Service of Ukraine [73]. (PDF) [file pone.0279783.s008.pdf]

S5 Table. Working hours of persons aged 15-70 years, Ukraine, 2021, in %.

	Women	Men	Total
Up to 19 hours	3.0	1.1	2.0
20–29 hours	7.0	3.9	5.3
30–39 hours	10.2	8.1	9.1
40 hours	69.9	71.0	70.5
More than 40 hours	9.9	15.9	13.1
Total	100	100	100

Sources: State Statistics Service of Ukraine [51].
