# Supplementary material for: High self-selection of Ukrainian refugees into Europe: Evidence from Kraków and Vienna
Source: PLoS One. 2023 Dec 20;18(12):e0279783. doi: 10.1371/journal.pone.0279783 (PMC10732457; doi:10.1371/journal.pone.0279783)
Supplement: S6 Table — Sources: UkrPL and UkrAiA. (PDF) [file pone.0279783.s009.pdf]

S6 Table. Usual working hours and plans for host country, persons aged 18-59 years, in %.

	Women		Men	
	Kraków	Vienna	Kraków	Vienna
**Usual working hours, if ever worked**				
1 to 9 hour(s)	20.9	10.7	(0.0)	3.8
10 to 19 hours	11.0	8.4	(10.0)	6.3
20 to 34 hours	9.9	16.0	(0.0)	15.2
35 or more hours	47.8	56.8	(60.0)	54.4
Seasonal work	0.8	0.7	(0.0)	1.3
Hours vary considerably	6.6	4.3	(30.0)	16.5
No answer	3.0	3.1	(0.0)	2.5
N	364	813	(10)	(79)
**Future plans within host country**				
Search for a job		72.2		70.8
To continue school/studying		5.2		7.3
Do not know		19.6		17.7
No answer		3.0		4.2
N		899		96
**Intended working hours, if wanting to work**				
Less than 20 hours		11.5		7.6
20 to 34 hours		49.3		26.6
35 or more hours		37.9		64.6
No answer		1.3		1.3
N		671		79

Sources: UkrPL and UkrAiA.
