# Supplementary material for: High self-selection of Ukrainian refugees into Europe: Evidence from Kraków and Vienna
Source: PLoS One. 2023 Dec 20;18(12):e0279783. doi: 10.1371/journal.pone.0279783 (PMC10732457; doi:10.1371/journal.pone.0279783)
Supplement: S7 Table — Intentions to stay in Country of Arrival (a) and to return to Ukraine (b), in %. Sources: UkrPL and UkrAiA. (PDF) [file pone.0279783.s010.pdf]

S7 Table. Intentions to stay in Country of Arrival (a) and to return to Ukraine (b), in %.

	(a) Do you plan to stay in Country of Arrival (CoA)?				(b) Which statements below may express your considerations about returning to Ukraine?					
	Stay in CoA	Not stay in CoA	Do not know	Chi^2-test	I have nothing to return to in Ukraine	I want to return as soon as the war ends	I may return in case the war ends	I may return even if the war continues	I do not have an idea, I do not know	Chi^2-test
Survey										
Kraków (Poland)	28	22	51		7	36	28	14	15	
Vienna (Austria)	47	9	44	***	8	34	25	6	27	***
Gender										
Male	55	9	36		16	34	24	6	20	
Female	40	13	47	**	7	35	26	8	24	*
Age										
Below 25	50	10	40		7	36	25	8	23	
25+	40	13	47	*	9	26	29	12	24	*
Residence before leaving Ukraine										
Kyiv	39	15	46		4	42	24	8	22	
Central Ukraine	41	12	47		11	35	24	10	21	
Western Ukraine	42	16	43		3	29	29	14	25	
Southern Ukraine	42	11	47		6	36	25	7	27	
Eastern Ukraine	42	10	47		17	28	28	4	22	***
Highest level of education										
Secondary general education or less	49	7	45		11	23	32	6	28	
Vocational education	37	17	45		7	34	25	7	27	
Bachelor degree	41	15	44		6	33	24	10	27	
Master degree or PhD	40	12	48	*	8	38	26	8	20	***
Skills in dominant language of CoA										
No skills in German/Polish language	39	13	48		8	36	25	8	23	
Skills in German/Polish language	50	11	38	**	7	29	29	9	26	
Relationship status										
Cohabiting or married partner is not in Ukraine	48	10	43		8	31	29	6	26	
Cohabiting or married partner is still in Ukraine	30	18	52		4	45	24	11	15	
Other family status: divorced, widowed, single (with or without partner)	47	10	43	***	11	27	26	7	29	***
Reasons for choice of CoA										
Unplanned, landed in CoA by chance	42	8	50		12	30	27	5	27	
Reason “unplanned, …” not reported	41	14	45	**	6	36	26	9	22	***
Family, friends or colleagues in CoA	41	13	45		6	35	27	9	23	
Reason “family or …” not reported	40	12	47		10	33	25	7	24	*
Easier to find work in CoA	70	1	28		13	21	31	4	30	
Reason “easier to …” not reported	40	13	47	***	7	35	26	9	23	*
Other reason reported (geographical proximity, welfare/health system)	35	17	48		8	39	21	12	20	

No other reason reported, ref.	43	12	46	**	8	33	27	7	25	**
No reason reported	11	26	63		8	58	8	17	8	
At least one reason reported	41	13	46	*	8	34	26	8	23	n.f.
N		1,547					1,540			

Sources: UkrPL and UkrAiA.

* $p < 0.05$, ** $p < 0.01$, *** $p < 0.001$. n.f. = Chi²-test not feasible (low cell count).
