# Supplementary material for: High self-selection of Ukrainian refugees into Europe: Evidence from Kraków and Vienna
Source: PLoS One. 2023 Dec 20;18(12):e0279783. doi: 10.1371/journal.pone.0279783 (PMC10732457; doi:10.1371/journal.pone.0279783)
Supplement: S8 Table — Confidence intervals of estimated coefficients (95%) for intentions to stay in Country of Arrival (a) and to return to Ukraine (b), average marginal effects shown in Table 2 in main text. Sources: UkrPL and UkrAiA. (PDF) [file pone.0279783.s011.pdf]

S8 Table. Confidence intervals of estimated coefficients (95%) for intentions to stay in Country of Arrival (a) and to return to Ukraine (b), average marginal effects shown in Table 2 in main text.

	(a) Do you plan to stay in Country of Arrival (CoA)?			(b) Which statements below may express your considerations about returning to Ukraine?				
	Stay in CoA	Not stay in CoA	Do not know	I have nothing to return to in Ukraine	I want to return as soon as the war ends	I may return in case the war ends	I may return even if the war continues	I do not have an idea, I do not know
Survey								
Kraków (Poland)	[-.24; -.14]	[.07; .17]	[.01; .14]	[-.04; .02]	[-.05; .07]	[.00; .11]	[.03; .11]	[-.17; -.08]
Vienna (Austria), ref.	-	-	-	-	-	-	-	-
Gender								
Male, ref.								
Female	[-.13; .04]	[-.10; .05]	[-.03; .17]	[-.10; -.02]	[-.16; .02]	[-.04; .13]	[-.08; .04]	[.02; .18]
Age								
Below 25	[-.09; .08]	[-.04; .08]	[-.11; .08]	[-.05; .04]	[-.10; .07]	[-.06; .10]	[.04; .14]	[-.16; -.01]
25+, ref.	-	-	-	-	-	-	-	-
Residence before leaving Ukraine								
Kyiv, ref.	-	-	-	-	-	-	-	-
Central Ukraine	[-.02; .14]	[-.11; .00]	[-.09; .08]	[.02; .11]	[-.14; .01]	[-.07; .07]	[-.04; .04]	[-.06; .07]
Western Ukraine	[-.03; .14]	[-.08; .04]	[-.12; .05]	[-.03; .04]	[-.23; -.07]	[-.04; .12]	[.00; .12]	[-.03; .11]
Southern Ukraine	[-.02; .11]	[-.11; -.01]	[-.05; .09]	[-.01; .05]	[-.12; .02]	[-.06; .07]	[-.05; .02]	[-.02; .10]
Eastern Ukraine	[-.01; .13]	[-.11; -.01]	[-.08; .08]	[.07; .16]	[-.19; -.05]	[-.03; .11]	[-.07; .00]	[-.06; .06]
Highest level of education								
Secondary general education or less	[-.02; .15]	[-.14; -.05]	[-.06; .12]	[-.01; .09]	[-.16; .00]	[-.02; .14]	[-.09; .02]	[-.05; .11]
Vocational education	[-.08; .09]	[-.06; .06]	[-.09; .09]	[-.03; .05]	[-.07; .09]	[-.07; .09]	[-.08; .01]	[-.07; .08]
Bachelor degree, ref.	-	-	-	-	-	-	-	-
Master degree or PhD	[-.06; .06]	[-.07; .02]	[-.04; .09]	[.00; .06]	[-.02; .09]	[-.03; .08]	[-.05; .03]	[-.13; -.02]
Skills in dominant language of CoA								
No skills in German/Polish language, ref.	-	-	-	-	-	-	-	-
Skills in German/Polish language	[.03; .16]	[-.06; .03]	[-.15; -.01]	[-.05; .03]	[-.11; .02]	[-.04; .08]	[-.05; .02]	[-.01; .10]
Relationship status								
Cohabiting or married partner is not in Ukraine, ref.	-	-	-	-	-	-	-	-
Cohabiting or married partner is still in Ukraine	[-.20; -.06]	[.02; .11]	[-.01; .13]	[-.06; .01]	[.08; .21]	[-.13; .00]	[.01; .08]	[-.17; -.05]
Other family status: divorced, widowed, single (with or without partner)	[-.06; .08]	[-.04; .05]	[-.08; .06]	[.00; .07]	[-.08; .05]	[-.11; .02]	[-.02; .05]	[-.04; .08]
Reasons for choice of CoA								
Unplanned, landed in CoA by chance	[-.11; .07]	[-.16; -.01]	[.01; .20]	[-.01; .08]	[-.11; .07]	[-.11; .07]	[-.07; .04]	[-.07; .09]
Reason "unplanned, …" not reported, ref.	-	-	-	-	-	-	-	-
Family, friends or colleagues in CoA	[-.14; .04]	[-.08; .04]	[-.02; .16]	[-.05; .04]	[-.05; .11]	[-.10; .06]	[-.02; .07]	[-.11; .04]
Reason "family or …" not reported, ref.	-	-	-	-	-	-	-	-
Easier to find work in CoA	[.14; .41]	[-.43; -.02]	[-.22; .12]	[-.01; .09]	[-.22; .04]	[-.07; .15]	[-.12; .05]	[-.05; .15]
Reason "easier to …" not reported, ref.	-	-	-	-	-	-	-	-
Other reason reported (geographical	[-.11; .05]	[-.08; -.04]	[-.03; .14]	[-.03; .05]	[-.01; .14]	[-.17; -.02]	[.00; .08]	[-.09; .05]

proximity, welfare/health system)								
No other reason reported, ref.	-	-	-	-	-	-	-	-
No reason reported	[-.70; -.04]	[-.10; .17]	[.03; .64]	[-.07; .23]	[-.06; .53]	[-.66; .16]	[-.04; .21]	[-.52; .22]
At least one reason reported, ref.	-	-	-	-	-	-	-	-

Sources: UkrPL and UkrAiA.

Note: Models additionally control for survey mode (paper-and-pencil or web survey), religious denomination, subjective health, employment status before leaving Ukraine and parenthood status.
