# Supplementary figures and images for: High self-selection of Ukrainian refugees into Europe: Evidence from Kraków and Vienna
Source: PLoS One. 2023 Dec 20;18(12):e0279783. doi: 10.1371/journal.pone.0279783 (PMC10732457; doi:10.1371/journal.pone.0279783)

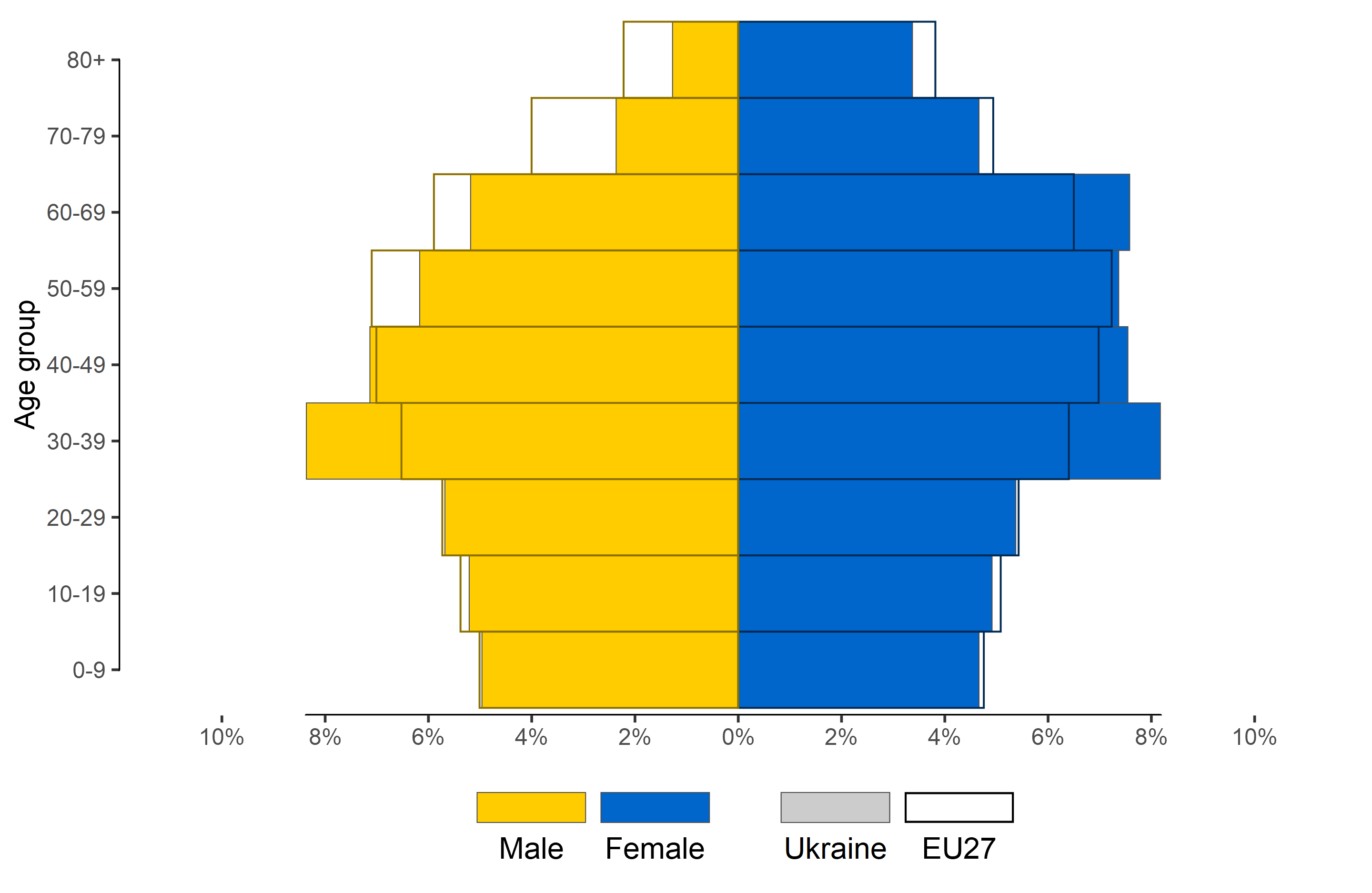

Supplement: S1 Fig — Sources: Eurostat [74]. UNFPA [75]. Note: Full bars show the Ukrainian population, whereas empty bars show the total EU-27 (composition of 2021) population. (TIF) [file pone.0279783.s012.tif]

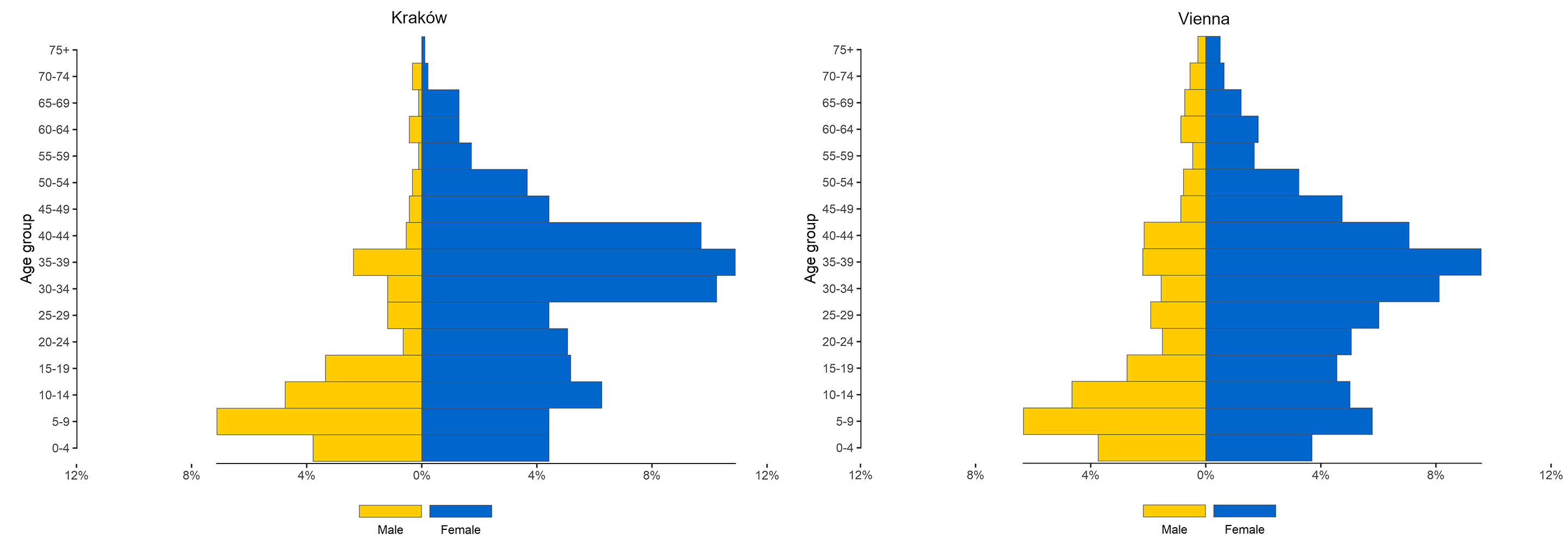

Supplement: S2 Fig — Sources: UkrPL and UkrAiA. Note: Figures include respondents as well as their children and partners if they stayed with them in Poland (N = 927) or Austria (N = 2,194), respectively. (TIF) [file pone.0279783.s013.tif]

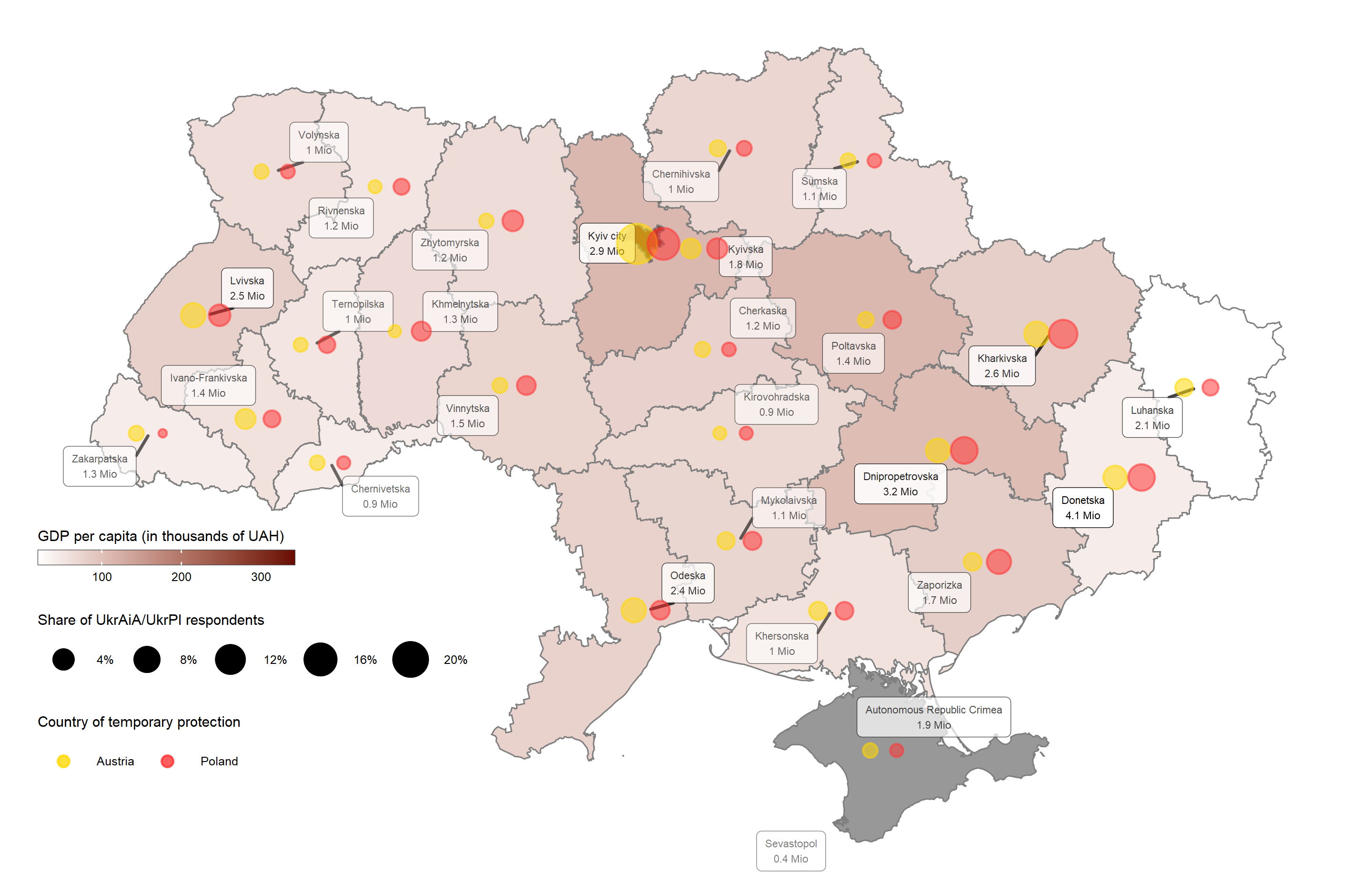

Supplement: S3 Fig — Sources: UkrPL and UkrAiA. UNFPA [52]. (TIF) [file pone.0279783.s014.tif]

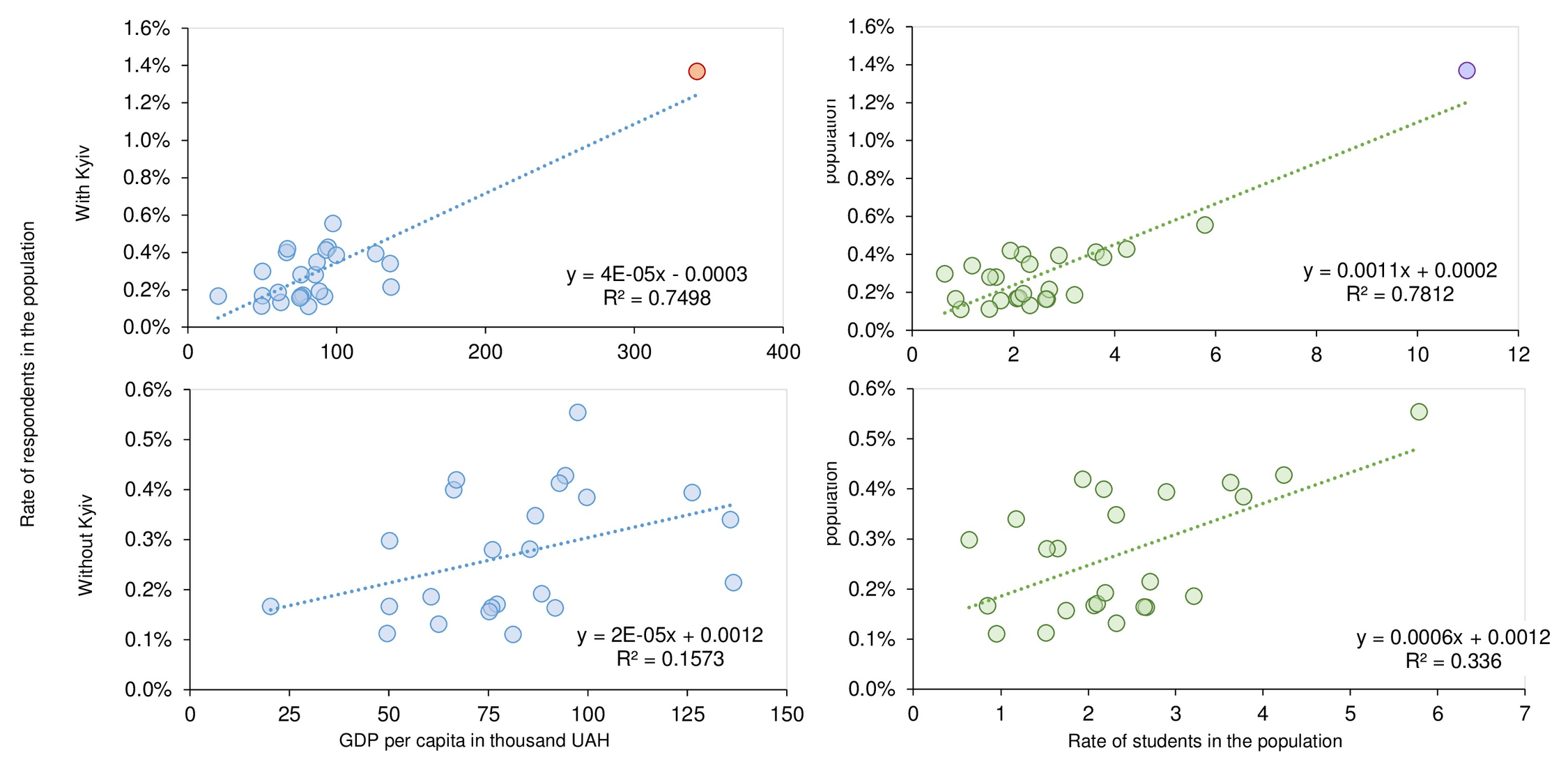

Supplement: S4 Fig — Correlation between respondents’ origin and GDP (left) as well as rate of students (right). Sources: UkrPL, UkrAiA and State Statistics Service of Ukraine [55]. Note: The rate of respondents in the population is calculated by dividing the number of respondents in the UkrPL/UkrAiA-survey originating from the region by the number of persons living in the region in 2021, multiplied by 100. The rate of students is calculated by dividing the number of students in the region by the number of persons living in the region in 2021, multiplied by 100. (TIF) [file pone.0279783.s015.tif]

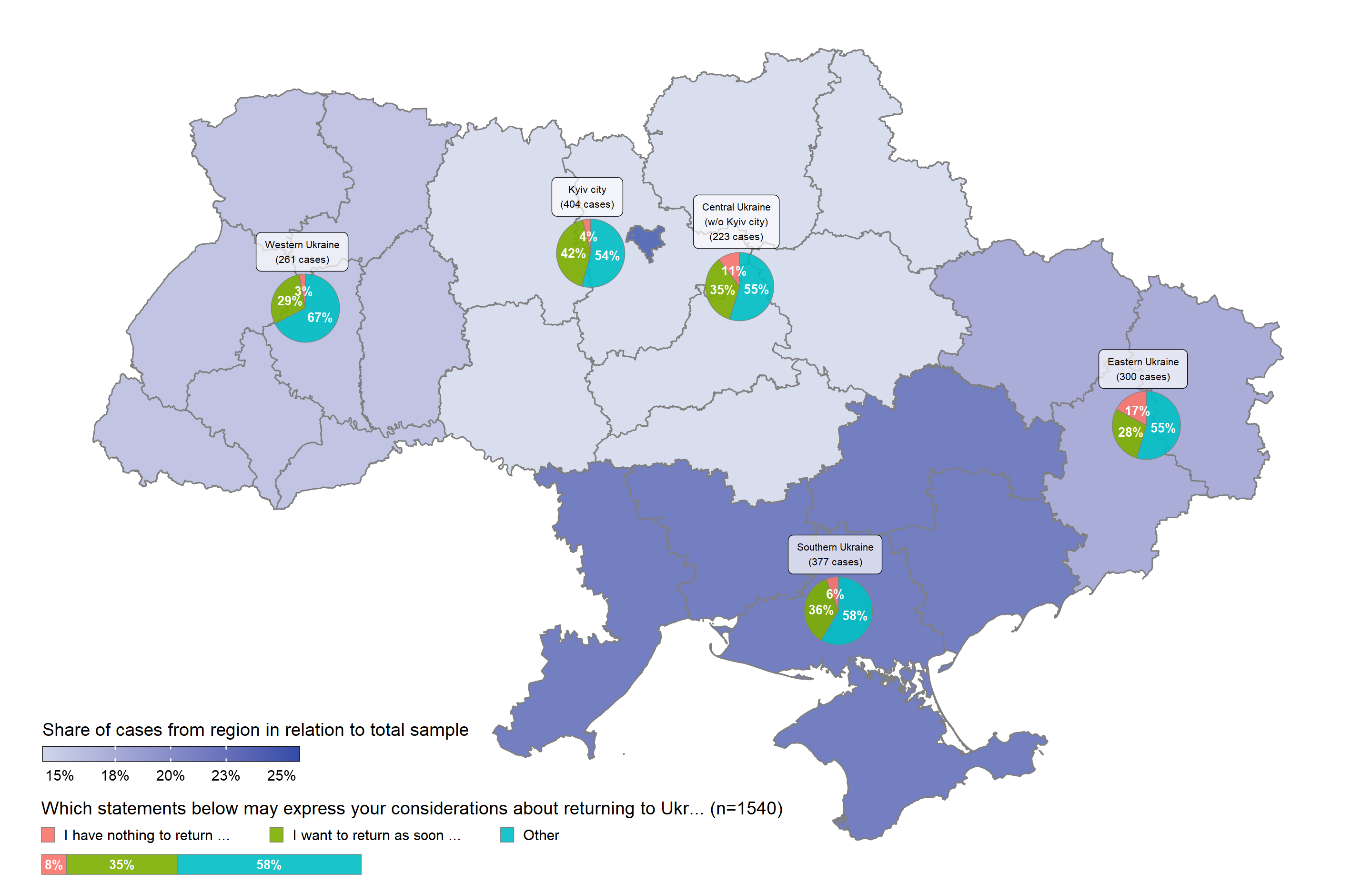

Supplement: S5 Fig — Sources: UkrPL and UkrAiA. See also S7 Table. (TIF) [file pone.0279783.s016.tif]
